# *Pax6* Inactivation in the Adult Pancreas Reveals Ghrelin as Endocrine Cell Maturation Marker

**DOI:** 10.1371/journal.pone.0144597

**Published:** 2015-12-11

**Authors:** Zeeshan Ahmad, Maria Rafeeq, Patrick Collombat, Ahmed Mansouri

**Affiliations:** 1 Max Planck Institute for Biophysical Chemistry, Department of Molecular Developmental Biology, RG Molecular Cell Differentiation, Goettingen, Germany; 2 Université de Nice Sophia Antipolis, Nice, France; 3 Inserm U1091, IBV, Diabetes Genetics Team, Nice, France; 4 JDRF, New York, NY, United States of America; 5 Genome and Stem Cell Center, GENKOK, Erciyes University, Kayseri, Turkey; 6 University of Goettingen, Department of Clinical Neurophysiology, Goettingen, Germany; INSERM UMRS 1138, FRANCE

## Abstract

The transcription factor *Pax6* is an important regulator of development and cell differentiation in various organs. Thus, *Pax6* was shown to promote neural development in the cerebral cortex and spinal cord, and to control pancreatic endocrine cell genesis. However, the role of *Pax6* in distinct endocrine cells of the adult pancreas has not been addressed. We report the conditional inactivation of *Pax6* in insulin and glucagon producing cells of the adult mouse pancreas. In the absence of *Pax6*, beta- and alpha-cells lose their molecular maturation characteristics. Our findings provide strong evidence that *Pax6* is responsible for the maturation of beta-, and alpha-cells, but not of delta-, and PP-cells. Moreover, lineage-tracing experiments demonstrate that *Pax6*-deficient beta- and alpha-cells are shunted towards ghrelin marked cells, sustaining the idea that ghrelin may represent a marker for endocrine cell maturation.

## Introduction

The pancreas develops from the endoderm at the foregut/midgut junction to give rise to two main cell compartments, comprising exocrine and endocrine cells. The major compartment of the pancreas is the exocrine tissue (acinar and duct cells) producing digestive enzymes that reach the duodenum through an intricate ductal system. The endocrine counterpart regroups hormone-producing cells into functional units called islets of Langerhans, consisting of beta-, alpha-, delta-, PP-, and epsilon-cells secreting insulin, glucagon, somatostatin, pancreatic polypeptide, and ghrelin, respectively, to control glucose homoeostasis. The development and maturation of the endocrine pancreas is under the control of a variety of transcription factors (for review see [1, 2]. Among these, members of the *Pax* gene family, *Pax4* and *Pax6*, were shown to play a crucial role in endocrine pancreas genesis [3–8]. Accordingly, *Pax4* inactivation resulted in the loss of beta- and delta-cells, concomitant with a proportional increase of glucagon-labeled cells [3]. Moreover, the forced expression of Pax4 in glucagon-producing alpha-cells of transgenic mice provoked their conversion into beta-like cells that counter chemically induced diabetes [9–12]. In contrast, the loss of *Pax6* gene activity was accompanied with a highly reduced number of all endocrine cells, and where glucagon-expressing cells were predominantly affected. Mice with conditional inactivation of *Pax6* in *Pdx1* or *Pax6* expression domains died few days postpartum and suffered from diabetes [5]. Of interest is the maintained expression of some pancreatic transcription factors such as Nkx2.2, Isl1, and Pdx1 in these *Pax6* mutant pancreata. This suggested that Pax6 is required to control the expression of genes involved in pancreatic endocrine function such as insulin, Glut2, and glucagon [5]. This is consistent with a recent report using global conditional inactivation of *Pax6* in 6 months old mice [7]. Several *in vitro* studies clearly confirmed that Pax6 is an important regulator of endocrine cell development and function. Accordingly, Pax6 was found to control the transcription of factors necessary for alpha-cell differentiation, such as proglucagon and the processing enzyme PC2 [13–15]. Along the same line of evidence Pax6 directly activates the transcription of MafB, cMaf, and Beta2/NeuroD, and Pax6 binding sites are found on their respective promoter [13–15]. However, Pax6 is not necessary for alpha-cell specification [16]. Interestingly, Pax6 also promotes proper beta-cell function, since it acts upstream of MafB which triggers the expression of the beta-cell maturation factors Pdx1 and MafA [13]. Similarly, Pax6 was shown to control the transcription of several factors that are crucial for beta-cell function, such as Glut2, PC1/3, Nkx6.1, and GLP1R [17]. Moreover, the knockdown of Pax6 function in primary beta-cells was accompanied by alterations in insulin synthesis and secretion, as well as decreased GLP-1 activity [17]. In addition, in zebrafish and in mice, pancreas specific enhancers that control *Pax6* gene activity were identified and were found to be dependent on Pbx and Meis homeoproteins [18, 19].

Notwithstanding, Pax6 function in distinct adult endocrine cells is still not clear. Here we report the conditional inactivation of *Pax6* in insulin- and glucagon-producing beta- and alpha-cells of adult mice, respectively. Using lineage tracing and endocrine hormone examination, we could show that beta- and alpha-cells lacking Pax6 function are shunted towards a ghrelin-labeled cell fate. Interestingly, beta- or alpha-cells with depleted *Pax6* gene activity fail to activate the expression of the maturation factors MafA or MafB, respectively, but maintained their respective pancreatic endocrine cell subtype destiny. While *Pax6-/-* beta-cells continue to express the transcription factor Pdx1, *Pax6*-deficient alpha-cells sustain the expression of the alpha-cell factor Arx. We conclude that Pax6 is necessary for the maintenance of molecular maturation characteristics of pancreatic endocrine cells.

## Materials and Methods

### Animals and treatments

*Pax6* ^*fl/fl*^ *;RIP-CreER* and *Pax6* ^*fl/fl*^ *;Glu-Cre* mice were generated by crossing *Pax6* ^*fl/fl*^ mice [20] with *RIP-CreERT* [21] and *Glucagon-Cre* mice [22], respectively. *Pax6* ^*fl/fl*^ *;TetO-Cre;Glu-rtTA* mice were generated by crossing *Pax6* ^*fl/fl*^ mice with *TetO-Cre* [23] and *Glucagon-rtTA* mice [11]. *Pax6* ^*fl/fl*^ *;RIP-CreER*, *Pax6* ^*fl/fl*^ *;Glu-Cre*, and *Pax6* ^*fl/fl*^ *;TetO-Cre;Glu-rtTA* mice were further crossed with *Rosa26-YFP* reporter mice [24] to incorporate the YFP reporter transgene for lineage tracing purpose. Lastly, *Pax6* ^*fl/fl*^ *;CG-CreER* mice were generated by crossing *Pax6* ^*fl/fl*^ mice with *CAG-CreERT* mice [25]. Genotyping was performed by means of PCR using genomic DNA isolated from tail-tip or ear biopsies. Primer sequences and PCR conditions are available on request.

**Fig 1:**
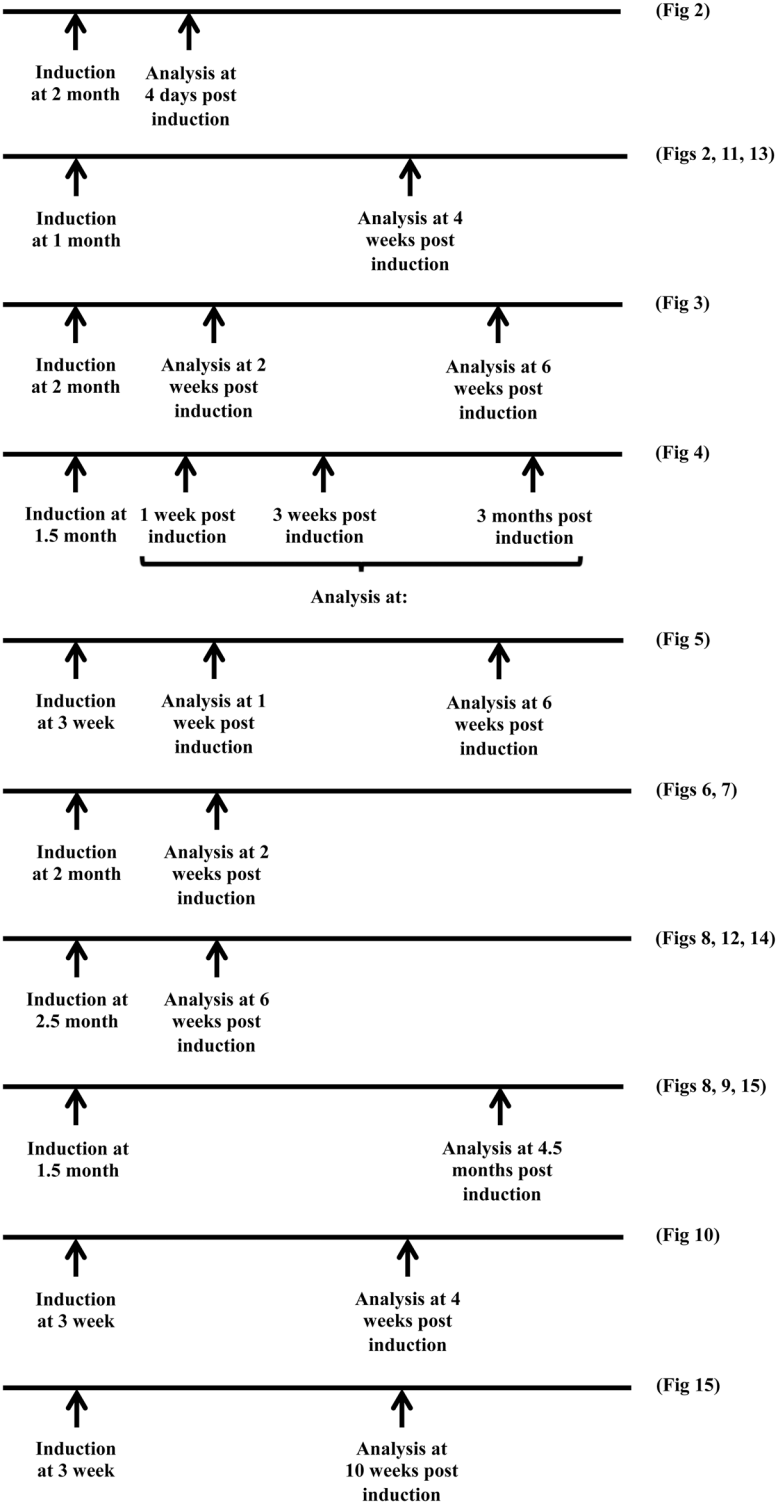
Different experimental time lines used for the beta-cell-specific *Pax6* ablation. Age at tamoxifen induction and analysis time point post-induction is indicated. After each timeline, the respective figure number is also mentioned.

Tamoxifen (Sigma-Aldrich) prepared in corn oil (at 20 mg/ml), was administered by intraperitoneal injection. In case of *RIP-CreERT* mice, 3–4 week old mice received 1 mg of tamoxifen per day for 3 days every other day, and 1.5–2.5 month old mice received 2 mg of tamoxifen per day for 4 days every other day. Age at tamoxifen-induction and pancreas-analyses time points used for beta-cell-specific *Pax6* ablation mice are shown in Fig 1 along with the respective figure numbers related to that specific setting. In case of *CAG-CreERT* mice, 2 month old mice were injected with 2 mg of tamoxifen per day for 5 consecutive days. Doxycycline (Sigma-Aldrich) was given to the mice in drinking water (at 2 g/l with 1% sucrose) for a period of 6 weeks. Doxycycline containing water was protected from light and replaced every week.

All animal experiments were conducted according to the German animal welfare law (LAVES Niedersachsen: Nds. Landesamt für Verbraucherschutz und Lebensmittelsicherheit (LAVES), Dezernat 33, 26029 Oldenburg). Hence, the experiments were performed according to the permission from Laves under: Vorhaben 33.9-42502-04-11/0402 (Kurztitel „Transgene01“) und 33.9-42502-04-11/0622 (Kurztitel „Transgene02“).

### Blood glucose measurements

Blood from the tail tip was used to measure the non-fasting blood glucose levels with the help of Accu-Chek Aviva (Roche) blood glucose meter.

### Immunohistochemistry

Mice were killed by CO_2_ inhalation and/or cervical dislocation. Pancreata were removed, washed in ice-cold PBS, and fixed in 4% paraformaldehyde (in PBS) for 1 hour at 4°C. Fixed pancreata were then washed in PBS (4x20minutes) and dehydrated in 25% sucrose (in PBS) for overnight at 4°C. Finally, they were embedded in the Jung Tissue Freezing Medium^™^ (Leica Microsystems). For immunofluorescence staining, 8 μm thick cryosections were used. Blocking was performed in 10% fetal calf serum (in PBS containing 0.1% Triton X-100) for 1 hour at room temperature. For some nuclear antigens, the signal was improved by an additional permeabilization with 0.2% Triton X-100 (in PBS) for 30 minutes at room temperature before the blocking. After blocking, the sections were incubated with primary antibodies (in blocking solution) for overnight at 4°C. This was followed by, washing in PBS, and incubation with appropriate secondary antibodies (in blocking solution) for 1 hour at room temperature. At the end, sections were washed again in PBS and mounted with Vectashield^®^ mounting medium containing DAPI as a nuclear counterstain.

The following primary antibodies were used in this study: guinea pig anti-insulin 1/1000, rabbit anti-somatostatin 1/600, and rat anti-Ki67 1/100 (Dako), mouse anti-insulin 1/500 (Sigma-Aldrich), mouse anti-glucagon 1/1000, rabbit anti-glucagon 1/50, rabbit anti-7B2 1/5000, and chicken anti-GFP 1/1000 (Abcam), rat anti-somatostatin 1/200, rabbit anti-pancreatic polypeptide (PP) 1/1000, rabbit anti-Rfx6 1/2000, rabbit anti-Arx 1/200, rabbit anti-Glut2 1/1000, rabbit anti-PC1/3 1/500, and rabbit anti-PC2 1/200 (Millipore), rabbit anti-MafA 1/500 and rabbit anti-MafB 1/1000 (Bethyl Labs), rabbit anti-C-peptide 1/100 (Cell Signaling), rabbit anti-IAPP 1/500 (Phoenix Pharma), goat anti-ghrelin 1/50 (Santa Cruz), rabbit anti-Pax6 1/300 (Covance), mouse anti-Nkx6.1 1/50 (Developmental Studies Hybridoma Bank), rabbit anti-Pdx1 1/2000 (kindly provided by C. Wright, Vanderbilt University, Nashville, TN), mouse anti-ghrelin 1/1000 (kindly provided by C. Tomasetto, Université Louis Pasteur, Strasbourg), rabbit anti-GLP-1 receptor 1/4000 (kindly provided by J.F. Habener, Harvard Medical School, MA), rabbit anti-7B2 1/200 and rabbit anti-ProSAAS 1/50 (kindly provided by I. Lindberg, University of Maryland-Baltimore, Maryland [26, 27]).

All of the secondary antibodies were obtained from Invitrogen (diluted at 1/1000) with the exception of sheep Cy5 anti-mouse (diluted at 1/300) that was obtained from Jackson Immunoresearch Laboratories. Images were acquired using either an Olympus BX60 fluorescent microscope or a Leica TCS SP5 confocal laser-scanning microscope. In case of triple immunofluorescence stainings, Cy5 channel was pseudo-colored in magenta, white, or green as required to show a better colocalization with other channels.

### Quantification of islet cell numbers and statistical analysis

For each quantification category, at least 3 mice per genotype per condition were used. The entire pancreata were serially cut and stained cells on every 20^th^ section were manually counted to obtain an average number per islet section. The values are depicted as mean±Standard error of mean (SEM). Statistical analysis was performed using student’s two-tailed t-test and p<0.05 was considered as significant.

## Results

### *Pax6* ablation in insulin-producing beta-cells of the adult pancreas

**Fig 2:**
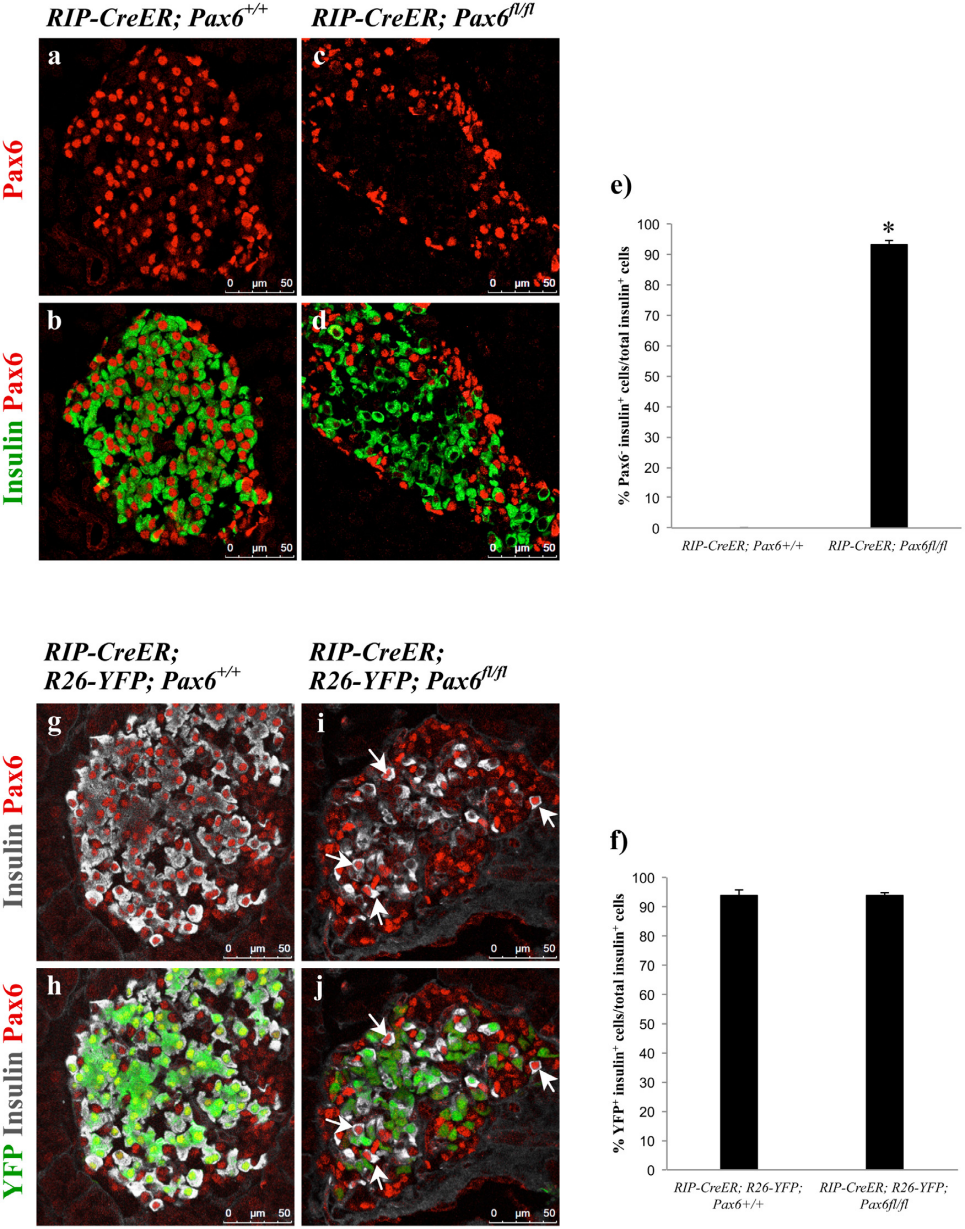
Beta-cell-specific ablation of *Pax6*. Double immunofluorescence staining of pancreatic cryosections from 2 month old mice at 4 days (a-d) or 4 weeks (g-j) after tamoxifen induction. *Pax6* expression is lost from a majority of insulin^+^ cells (c, d). Ablation of *Pax6* is specific to YFP labeled cells (i, j) as YFP^-^ insulin^+^ cells continue to express *Pax6* (arrows i, j). Quantification of *Pax6* ^-^ insulin^+^ (e) and YFP^+^ insulin^+^ (f) cells in the pancreata of 2 month old mice at 4 days after tamoxifen induction (n = 3). Error bars represent SEM; *p<0.05.

Pancreatic endocrine cell function was shown to depend on *Pax6* gene activity. In the absence of *Pax6* the number of the cells expressing insulin and glucagon was altered [4, 7, 8]. However, the role of Pax6 in specific endocrine cell types in the adult pancreas is still obscure. We therefore used *Pax6* ^*fl/fl*^ mice and *RIP-CreERT* transgenic mice to generate animals where *Pax6* is specifically inactivated in beta-cells upon tamoxifen induction (*RIP-CreERT;Pax6* ^*fl/fl*^). Furthermore, we used lineage tracing to follow the fate of *Pax6*-deficient beta-cells over an extended time period. Following tamoxifen administration, the successful depletion of Pax6 in beta-cells was confirmed by immunohistochemical analyses showing 95% KO efficiency (Fig 2).

Previous studies revealed that *Pax6* loss-of-function in mouse embryonic, as well as adult, pancreas leads to hyperglycemia [5, 7]. Similarly, our adult beta-cell-specific *Pax6* knockout mice developed hyperglycemia with blood glucose levels reaching as high as 450 mg/dl (see later). This augmentation in blood glucose level was associated with a decrease in insulin positive cell numbers in the pancreas of mutant mice (see below).

### Depletion of *Pax6* gene activity provokes the loss of mature beta-cell molecular characteristics and the activation of ghrelin expression

**Fig 3:**
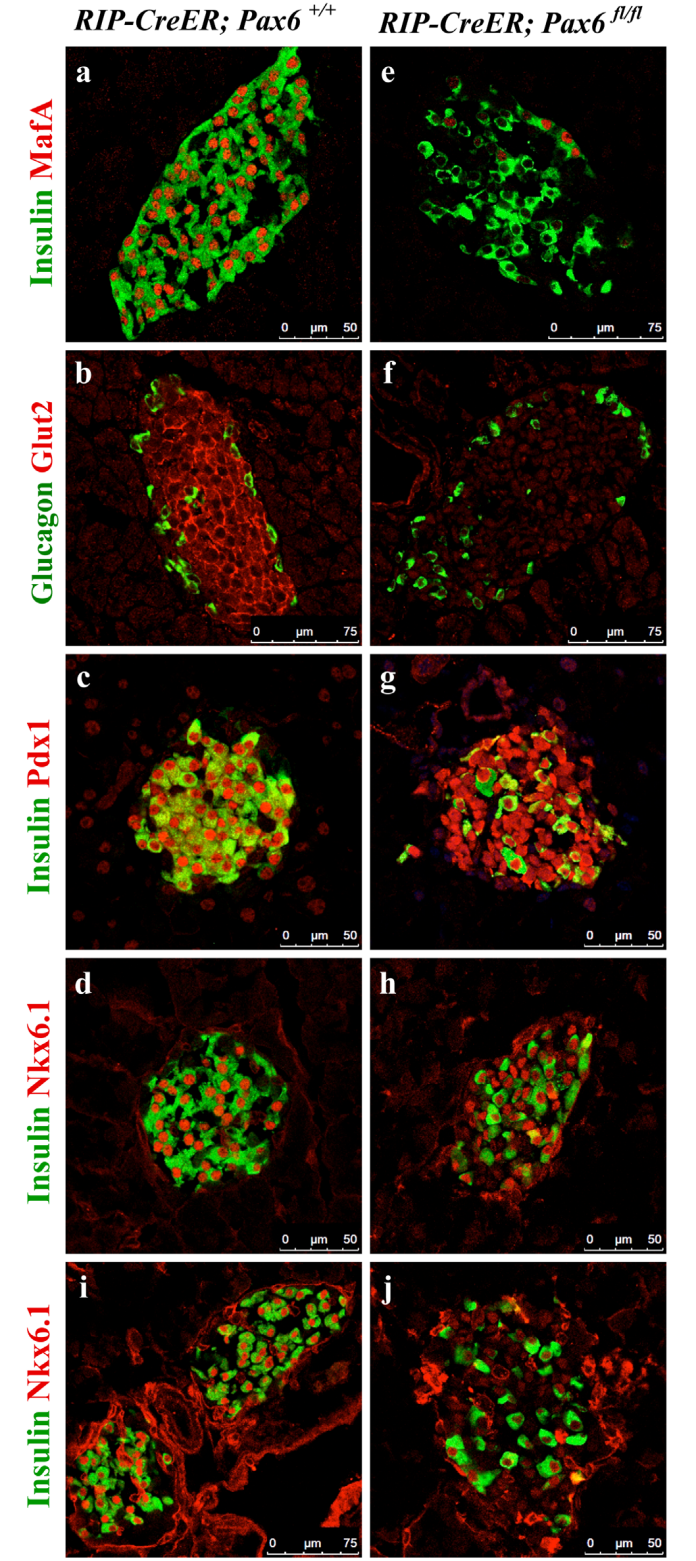
Expression of beta-cell-related factors in the islets of beta-cell-specific *Pax6* KO mice. Double immunofluorescence staining of pancreatic cryosections from 2.5 month old mice at 2 weeks after tamoxifen induction (a-h) or 3.5 month old mice at 6 weeks after tamoxifen induction (i, j). Expression of MafA and Glut2 is lost (e, f), expression of Pdx1 is maintained (g), and that of Nkx6.1 is maintained at 2 weeks (h) but sharply reduced at 6 weeks (j) post-tamoxifen induction in the islets of beta-cell-specific *Pax6* KO pancreata.

**Fig 4:**
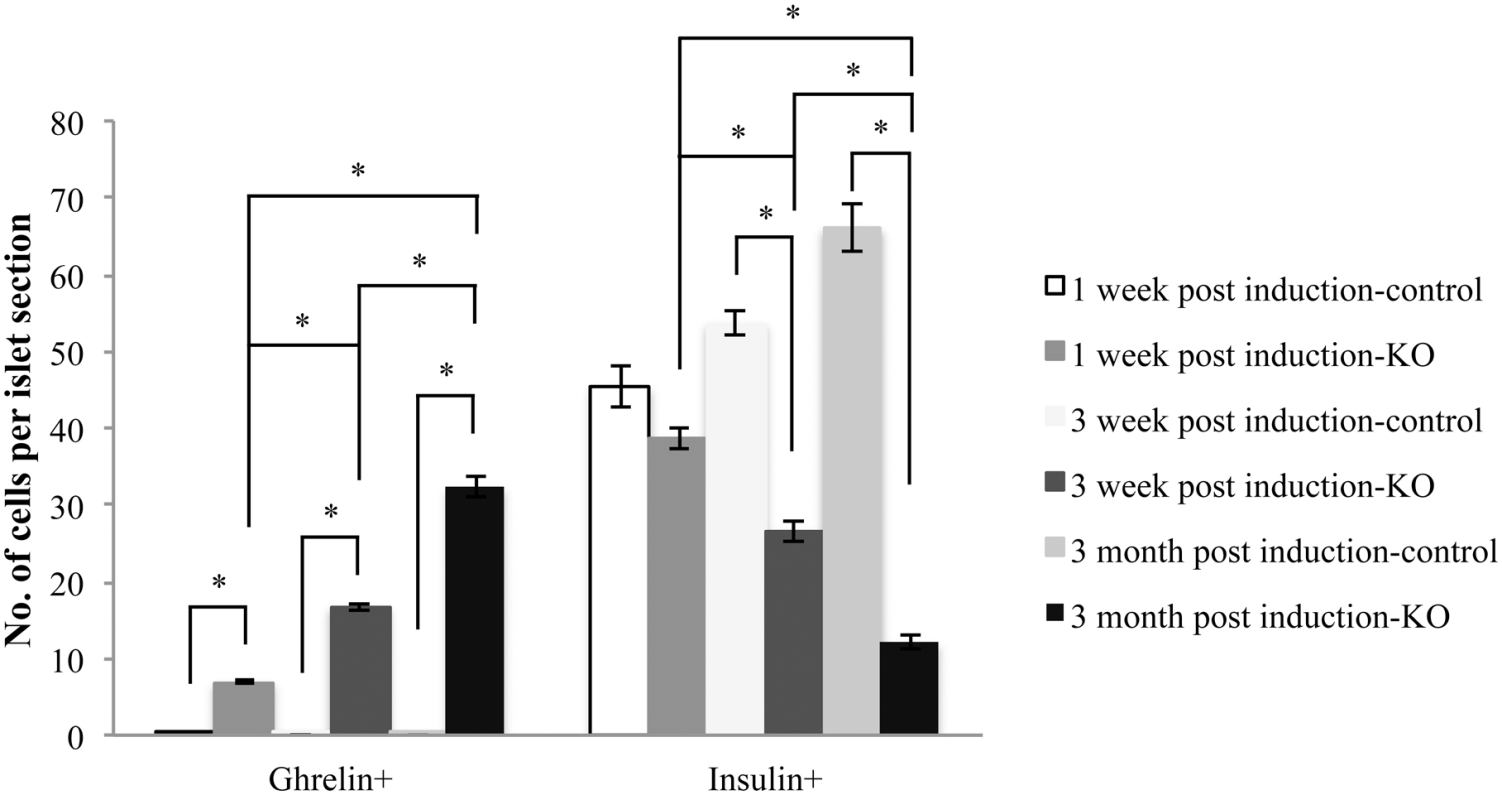
Inverse relationship of ghrelin and insulin expression in the pancreata of beta-cell-specific *Pax6* KO mice. Quantification of ghrelin^+^ and insulin^+^ cells in the islets of beta-cell-specific *Pax6* KO mice (Control = *RIP-CreER;Pax6* ^*+/+*^, KO = *RIP-CreER;Pax6* ^*fl/fl*^) injected at 1.5 month of age and analysed at different indicated time points post tamoxifen induction (n = 3). Error bars represent SEM; *p<0.05.

**Fig 5:**
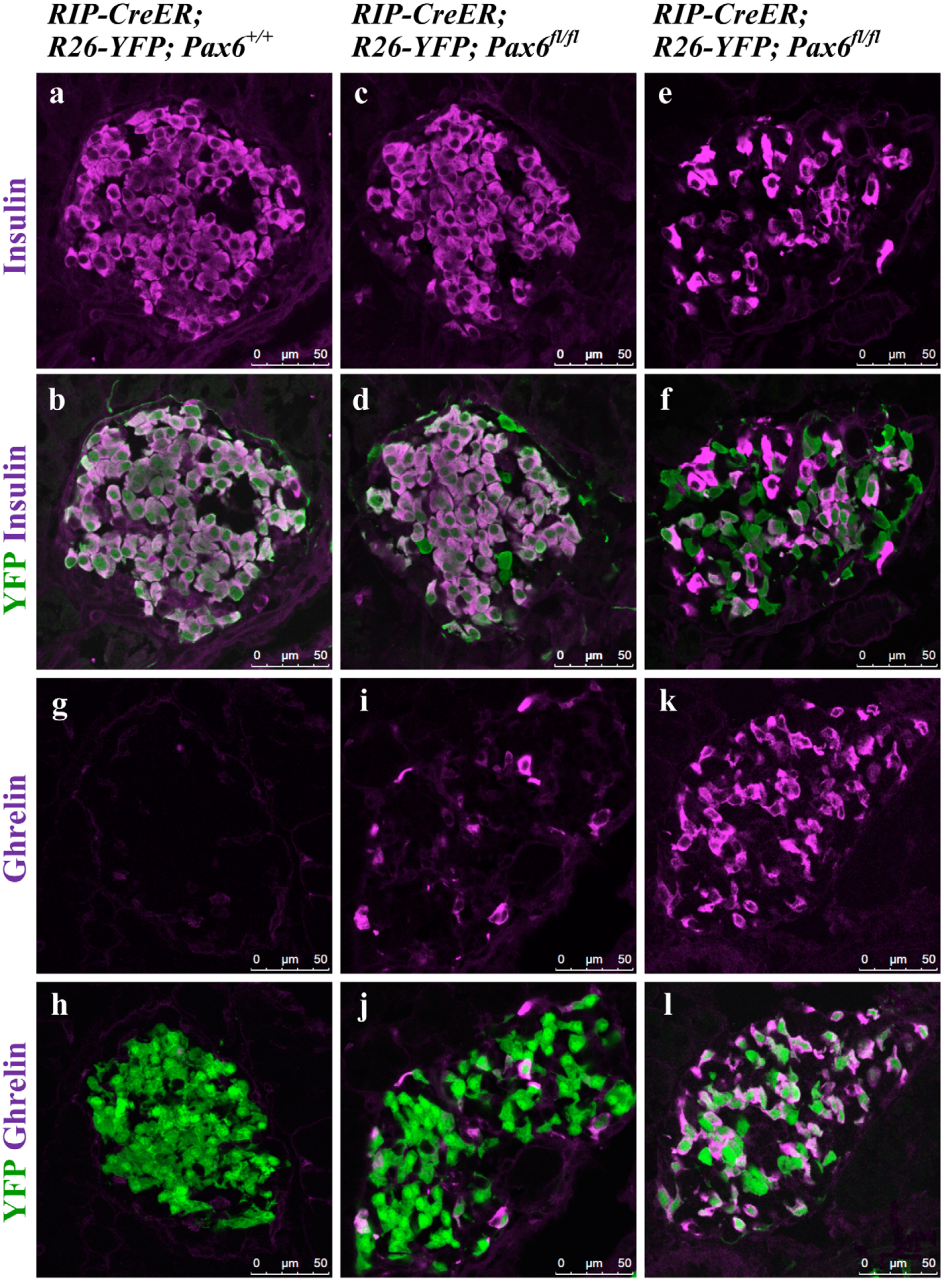
Gradual decrease in the population of insulin expressing cells and a proportional augmentation in the population of ghrelin expressing cells in the islets of beta-cell-specific *Pax6* KO mice. Immunofluorescence staining of pancreatic cryosections from 4 week old mice at 7 days after tamoxifen induction (a-d and g-j) and 9 week old mice at 6 weeks after tamoxifen induction (e, f and k, l). (a, b) In the control islets, all of the YFP^+^ cells express insulin. (c, d) At 7 days after tamoxifen induction few YFP^+^ *Pax6*-deficient cells are negative for insulin expression. (e, f) At 6 weeks after tamoxifen induction a majority of the YFP^+^ *Pax6*-deficient cells are negative for insulin expression. (g, h) Ghrelin^+^ cells are not detected in the control islets. (i, j) At 7 days after tamoxifen induction few YFP^+^ *Pax6*-deficient cells express ghrelin. (k, l) At 6 weeks after tamoxifen induction a majority of the YFP^+^ *Pax6*-deficient cells express ghrelin.

**Fig 6:**
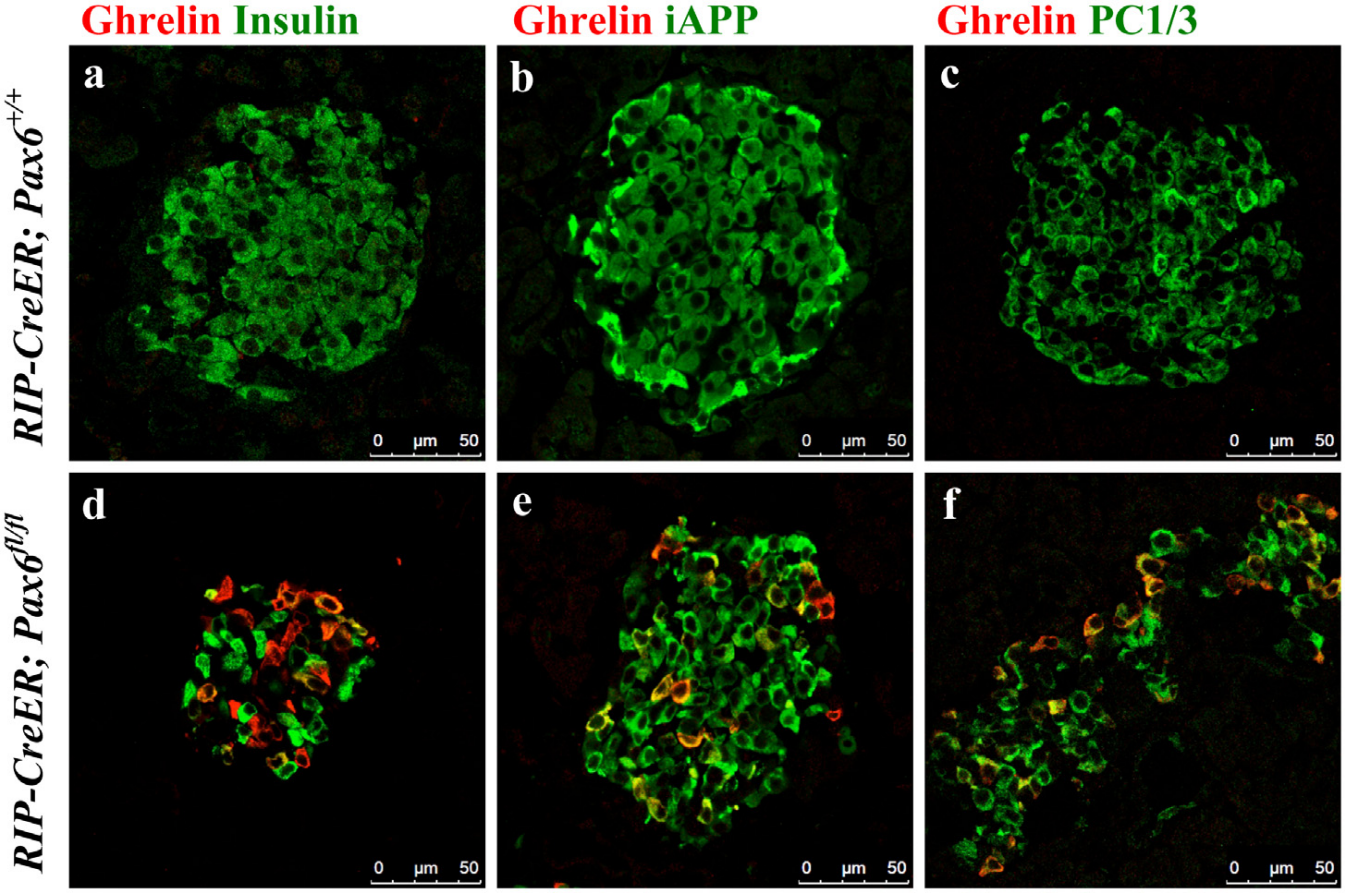
Co-expression of ghrelin with insulin, iAPP, and PC1/3 in the islets of beta-cell-specific *Pax6* KO mice. Double immunofluorescence staining of pancreatic cryosections from 2.5 month old mice at 2 weeks after tamoxifen induction. Ghrelin^+^ cells are not detected in the control islets (a-c). In the beta-cell-specific *Pax6* KO islets, ghrelin expression is up regulated (d-f) and some of the ghrelin^+^ cells co-express insulin (d). Additionally, these ghrelin^+^ cells co-express iAPP (e) and PC1/3 (f).

**Fig 7:**
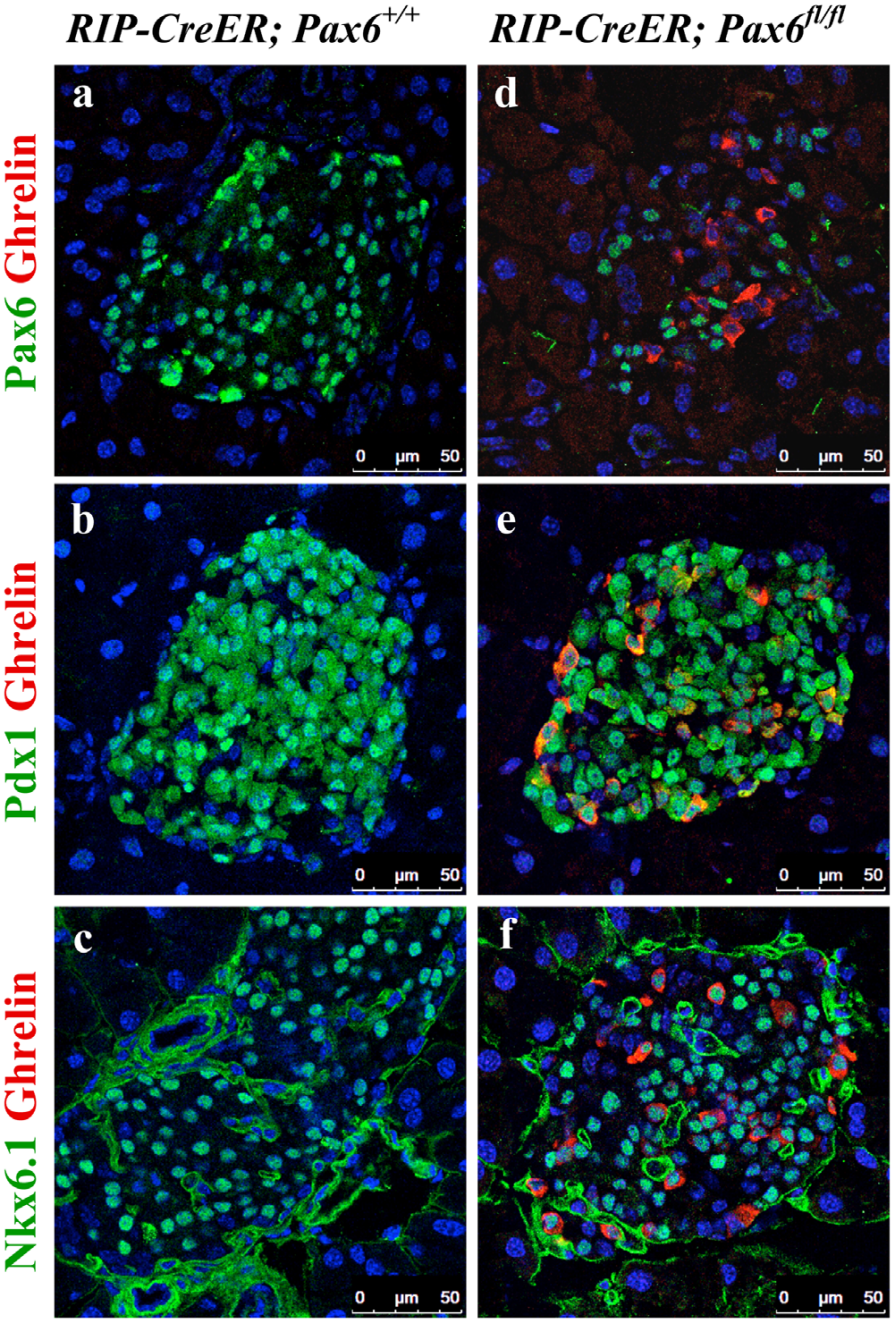
Expression of beta-cell-related transcription factors in the ghrelin^+^ cell population of beta-cell-specific *Pax6* KO islets. Double immunofluorescence staining of pancreatic cryosections from 2.5 month old mice at 2 weeks after tamoxifen induction. Ghrelin expression is not detected in the control islets (a-c). In beta-cell-specific *Pax6* KO islets, ghrelin expression is up regulated and ghrelin^+^ cells are negative for *Pax6* (d) but positive for Pdx1 (e), and Nkx6.1 (f).

Following two weeks of tamoxifen induction, insulin-producing beta-cells lost the expression of MafA and Glut2, two important markers of mature beta-cells (Fig 3). However, while the expression of Pdx1 remained unaffected, that of Nkx6.1 first appeared normal few days past-tamoxifen treatment, and gradually decreased at later time points (Fig 3d, 3h, 3i and 3j). Supporting this result, a qRT-PCR performed at 3 months post-tamoxifen induction also showed decrease in the expression of *MafA*, *Glut2*, and *Nkx6*.*1*. However, the expression of *Pdx1* as well as that of *Nkx2*.*2* was not significantly changed (S1 Fig). In addition, when followed over a longer time-period, it was possible to notice a decrease in the number of insulin positive cells in the KO pancreata with a concomitant augmentation in the content of ghrelin labeled cells (Figs 4 and 5). Of interest was the detection of some ghrelin/insulin co-positive cells (Fig 6). A decrease in the expression of *insulin* and augmentation in the expression of *ghrelin* was also evident by qRT-PCR (S1 Fig). Collectively, these observations are consistent with the idea that, following *Pax6* inactivation, beta-cells lose insulin expression and start to express ghrelin instead. This was supported by the fact that these ghrelin expressing cells were positive for beta-cell-specific transcription factors, such as Pdx1 and Nkx6.1 (Fig 7). Additionally, they also expressed PC1/3 and iAPP, two other beta-cell-related factors (Fig 6). It is important to notice that iAPP production was never affected in *Pax6*-deficient beta-cells. As a consequence, the normal iAPP/ insulin ratio is altered and might contribute to further deterioration of beta-cell function due to the formation of amyloid deposits [28]. Moreover, in the islets of beta-cell-specific *Pax6* knockout mice the numbers of glucagon^+^, somatostatin^+^, and PP^+^ cells were also increased (S2 Fig).

### Lineage tracing revealed that *Pax6*-/- beta-cells are shunted towards ghrelin-labeled cells

**Fig 8:**
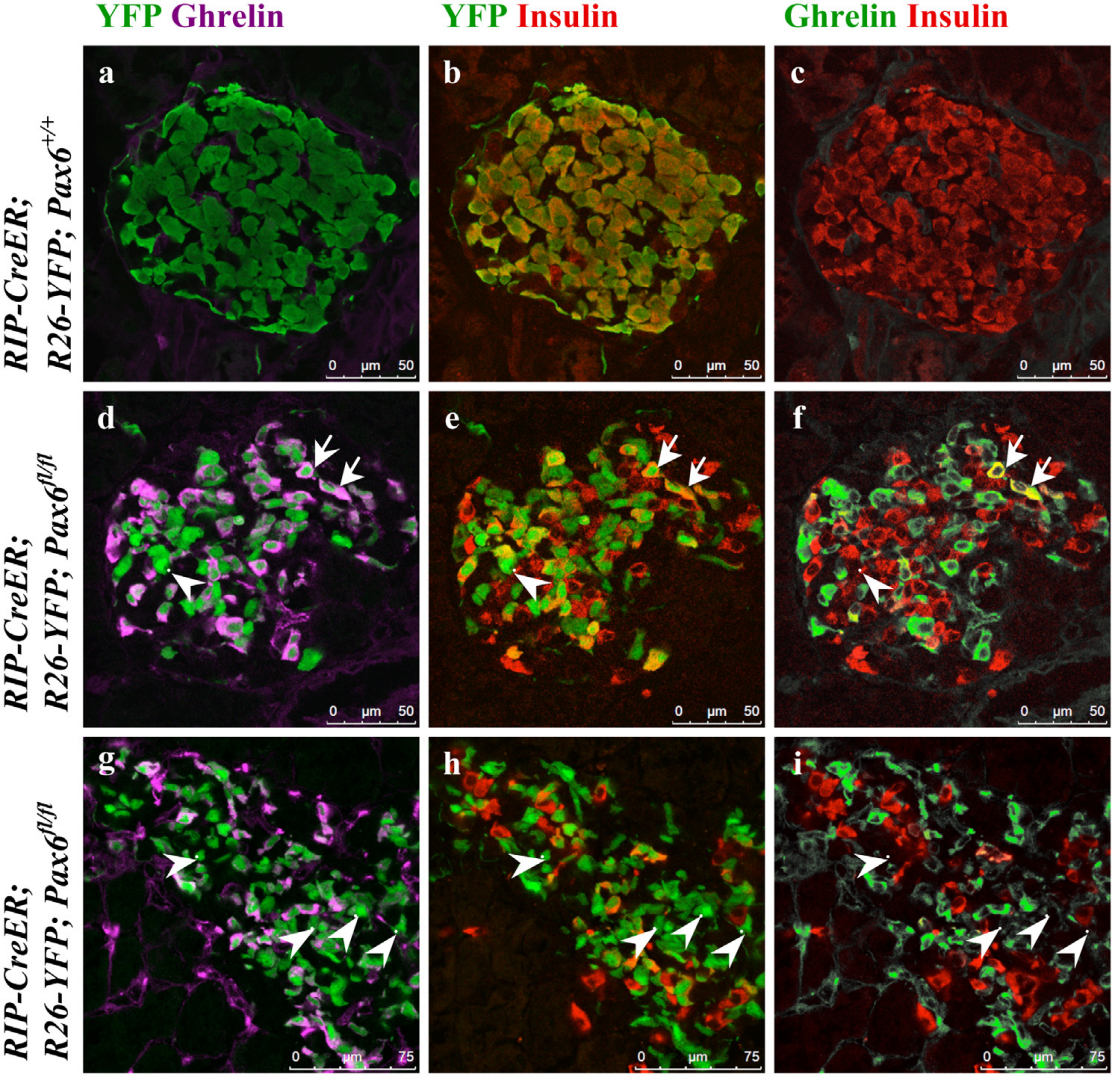
Ghrelin cells originate from beta-cells in the beta-cell-specific *Pax6* KO islets. Double immunofluorescence staining of pancreatic cryosections from 4 month old mice at 6 weeks after tamoxifen induction (a-f) and 6 month old mice at 4.5 months after tamoxifen induction (g-i). Ghrelin expression is not detected in the control islets (a-c). In beta-cell-specific *Pax6* KO islets, ghrelin expression is up regulated and that of insulin down regulated in the YFP labeled *Pax6*-deficient cells (d-f). Most of the ghrelin^+^ cells have lost the expression of insulin but some are still insulin^+^ (arrows d-f). Some YFP^+^ cells in the KO islets are negative for both insulin and ghrelin (arrowhead d-f). At 4.5 months after tamoxifen induction many of the YFP^+^ cells are negative for both insulin and ghrelin (arrowheads g-i).

**Fig 9:**
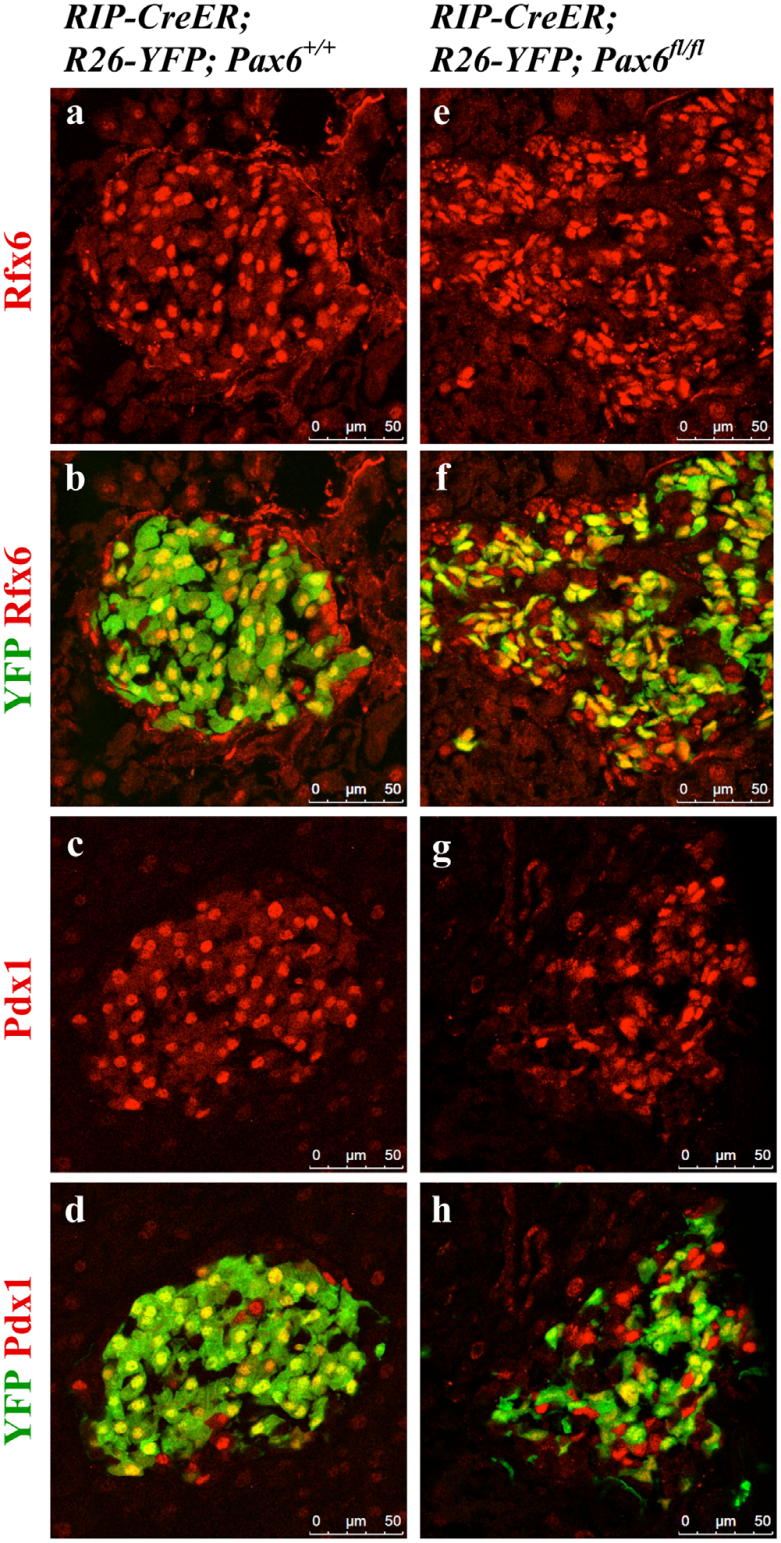
Expression of Rfx6 and Pdx1 in the islets of beta-cell-specific *Pax6* KO mice. Immunofluorescence staining of pancreatic cryosections from 6 month old mice at 4.5 month after tamoxifen induction. Expression of Rfx6 (e, f) and Pdx1 (g, h) is maintained in the YFP^+^ *Pax6*-deficient cells of the beta-cell-specific *Pax6* KO islets.

Lineage tracing with YFP reporter clearly demonstrated the conversion of beta-cells into ghrelin-expressing cells. Indeed, the analysis of KO pancreata (*RIP-CreERT;Rosa26-YFP;Pax6* ^*fl/fl*^) at different time points following tamoxifen induction revealed a progressive conversion of reporter-labeled insulin positive cells into YFP-marked ghrelin positive cells (Figs 5 and 8). Additionally, some of the reporter-labeled ghrelin positive cells lose the expression of ghrelin over time, as evidenced by the presence of reporter labeled insulin and ghrelin negative (YFP^+^ insulin^-^ ghrelin^-^) cells. Of interest was the observation that the YFP-labeled cells maintain the expression of Rfx6 and Pdx1 even at 4.5 month after tamoxifen induction. We conclude that in the absence of Pax6 activity adult beta-cells maintain their insulin-producing destiny, but lose the molecular characteristics of mature beta-cells (Fig 9).

**Fig 10:**
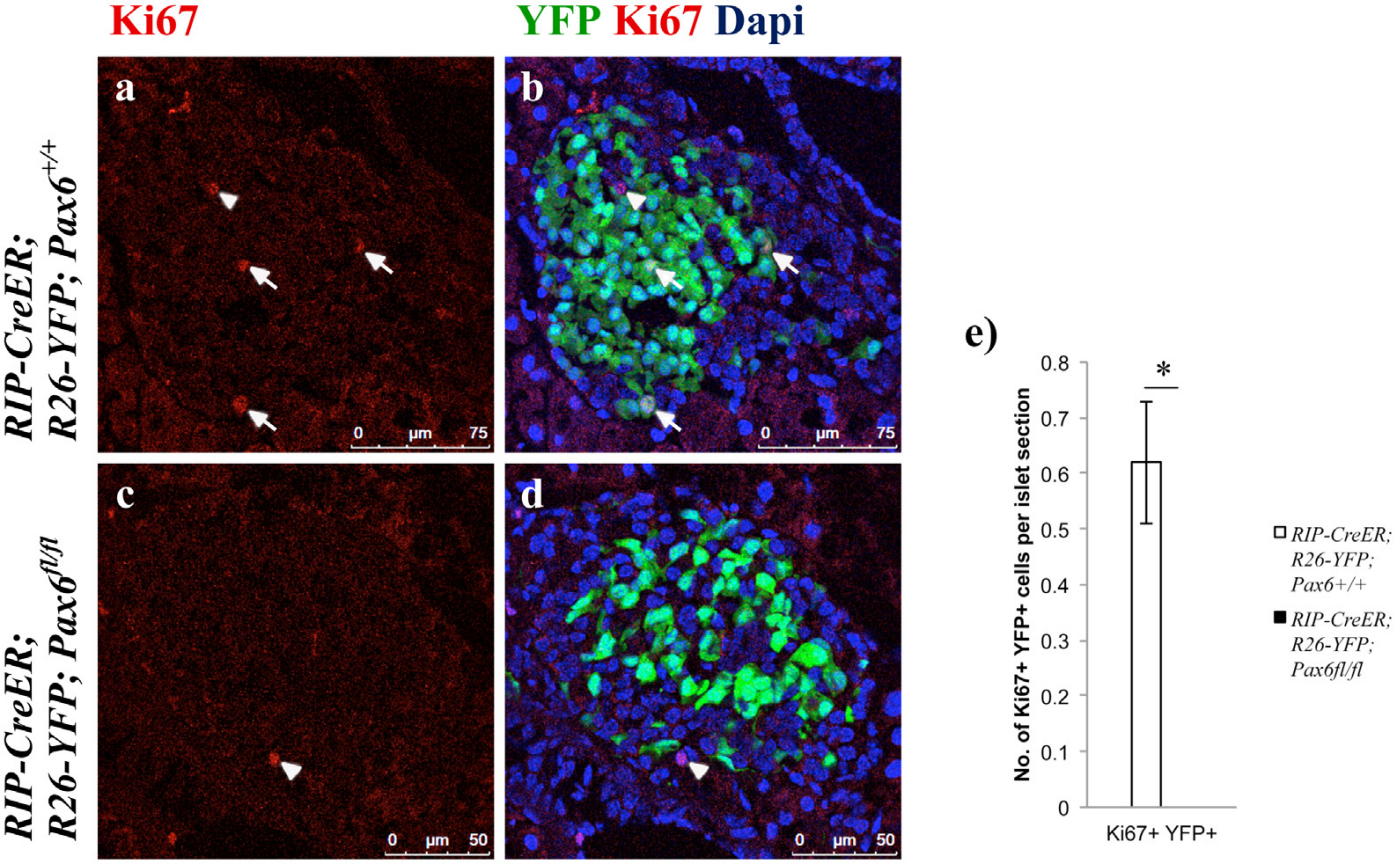
*Pax6*-deficient beta-cells do not proliferate. Immunofluorescence staining of pancreatic cryosections from 7 week old mice at 4 weeks after tamoxifen induction. Ki67^+^ YFP^+^ cells are detected in the control islets (arrows a, b) but not in the beta-cell-specific *Pax6* KO islets (c, d). On the other hand, Ki67^+^ YFP^-^ cells are detected in both the control and KO islets (arrowhead a-d). Quantification of Ki67^+^ YFP^+^ cells (e) in the islets of 7 week old mice at 4 weeks after tamoxifen induction (n = 3). Error bars represent SEM; *p<0.05.

We further noticed that the numbers of YFP-labeled cells in beta-cell-specific *Pax6*-deficient islets were always significantly inferior to those found in control islets. We therefore checked cell survival and cell proliferation in control and mutant islets. While apoptosis of insulin producing cells was not affected (S3 Fig), their proliferation was indeed defective. At 7 weeks of age (4 weeks post-tamoxifen induction), YFP^+^ Ki67^+^ cells could not be detected in the beta-cell-specific *Pax6*-deficient islets. However, such double positive cells were present in the control islets (Fig 10). It appears that Pax6 depletion may provoke decreased cell proliferation.

### *Pax6*-deficiency may affect insulin-processing machinery

**Fig 11:**
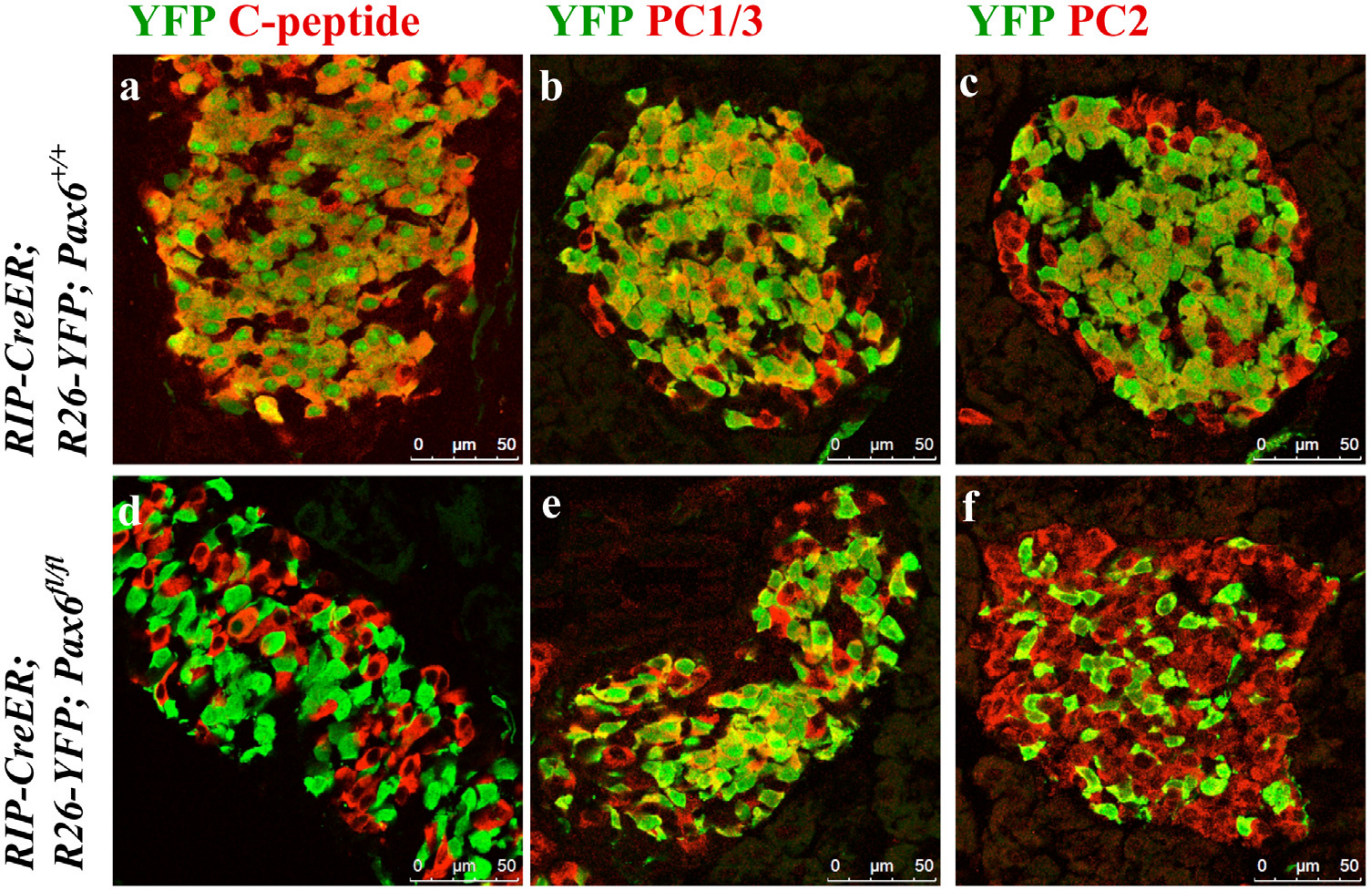
Expression of C-peptide, PC1/3, and PC2 in the islets of beta-cell-specific *Pax6* KO mice. Double immunofluorescence staining of pancreatic cryosections from 2 month old mice at 4 weeks after tamoxifen induction. In beta-cell-specific *Pax6* KO islets, C-peptide expression is lost from majority of the YFP^+^ *Pax6*-deficient cells (d). However, PC1/3 (e) and PC2 (f) expression is maintained in the YFP^+^ *Pax6*-deficient cells.

**Fig 12:**
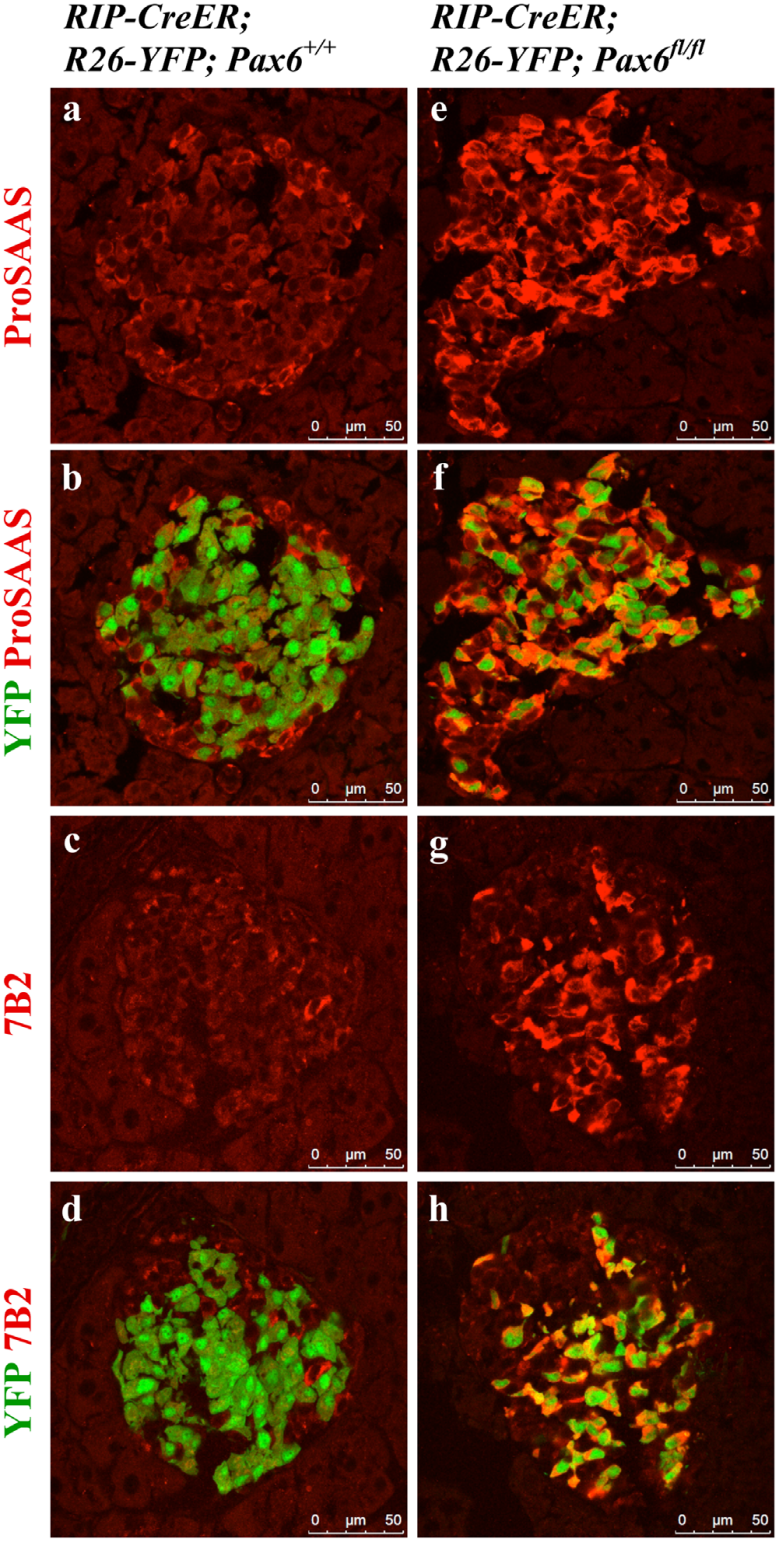
Expression of proSAAS and 7B2 in the islets of beta-cell-specific *Pax6* KO mice. Double immunofluorescence staining of pancreatic cryosections from 4 month old mice at 6 weeks after tamoxifen induction. In the control islets, expression of proSAAS and 7B2 is low in the YFP labeled beta-cells (a-d). In beta-cell-specific *Pax6* KO islets, expression of both proSAAS and 7B2 is highly up regulated in the YFP^+^ *Pax6*-deficient cells (e-h).

Pax6 was shown to directly down regulate Pcsk1n expression and thereby to affect pro-insulin processing [29]. To analyse whether *Pax6* inactivation has an impact on insulin processing and release, we examined the expression of c-peptide, PC1/3, PC2, proSAAS, and 7B2. In *Pax6*-deficient insulin-producing cells the expression of c-peptide was lost before that of insulin suggesting a defect in insulin processing. However, the expression of pro-hormone convertases 1/3 and 2 that are required for such process was normal (Fig 11 and S1 Fig). On the other hand, expression of proSAAS and 7B2, the regulatory peptides for PC1/3 and PC2, respectively, was increased (Fig 12). Elevated expression of proSAAS is known to inhibit the activity of PC1/3 supporting the speculation that insulin processing may indeed be disturbed. In contrast, the significance of increased 7B2 expression is still not clear.

**Fig 13:**
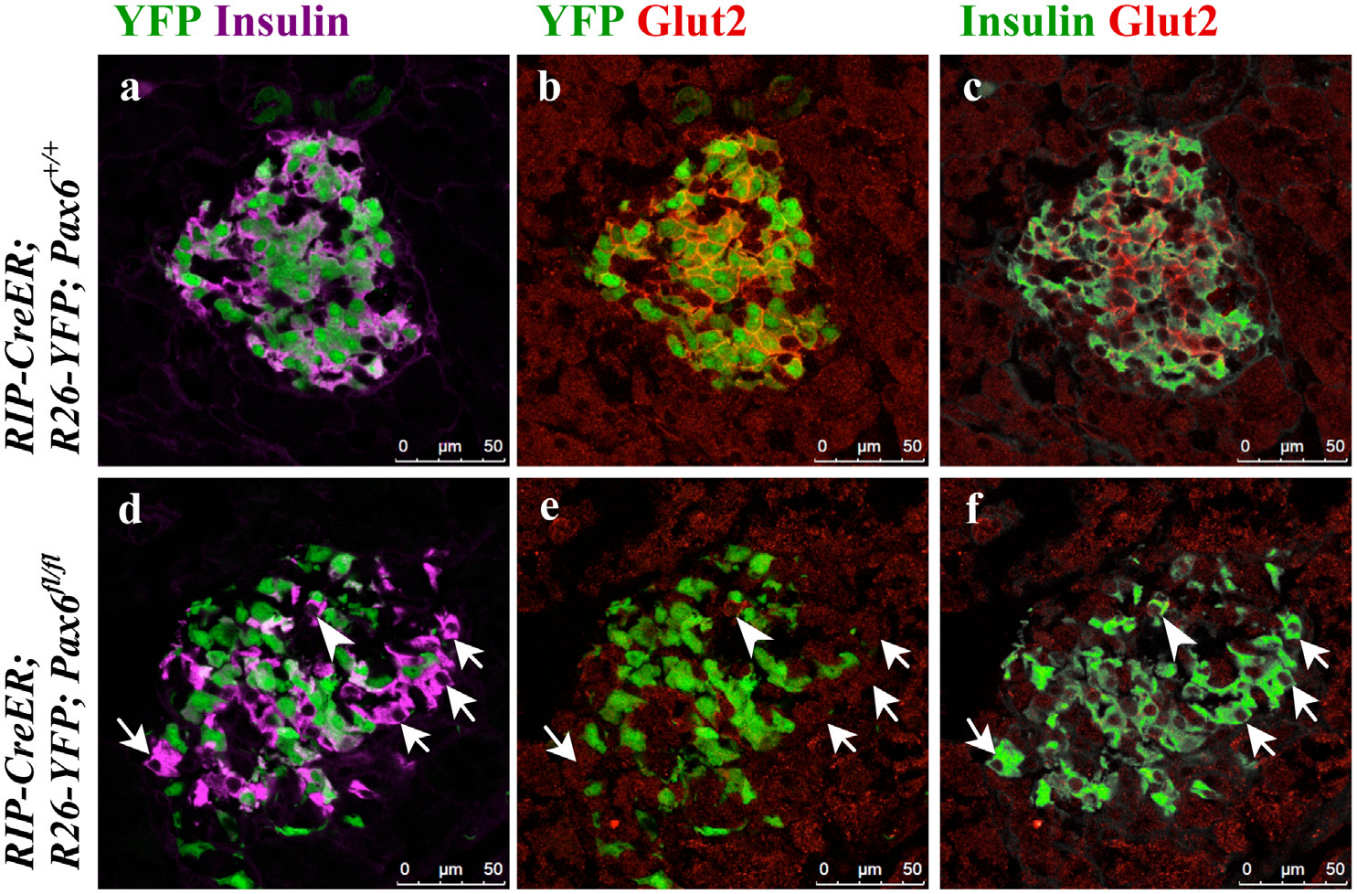
Loss of Glut2 expression due to direct and/or indirect effect of *Pax6* ablation in the islets of beta-cell-specific *Pax6* KO mice. Double immunofluorescence staining of pancreatic cryosections from 2 month old mice at 4 weeks after tamoxifen induction. In the control islets, expression of Glut2 is detected in both YFP^+^ and YFP^-^ insulin^+^ cells (a-c). In the beta-cell-specific *Pax6* KO islets, Glut2 expression is lost in the YFP labeled *Pax6*-deficient cells as well as in a majority of the YFP^-^ insulin^+^ cells (arrows d-f). Rarely, some YFP^-^ insulin^+^ cells do express Glut2 in the KO islets (arrowhead d-f).

**Fig 14:**
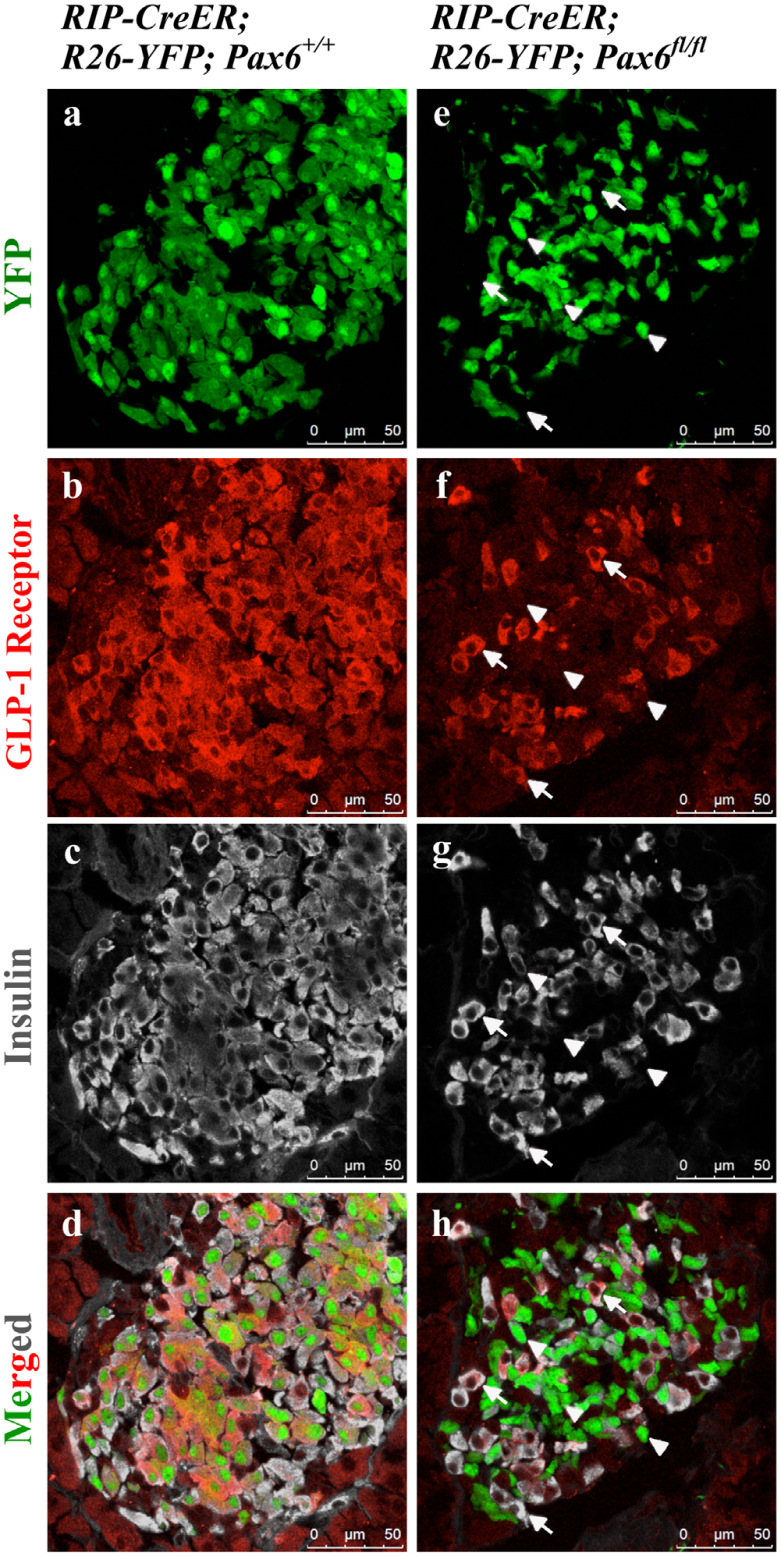
Expression of GLP-1 receptor in the islets of beta-cell-specific *Pax6* KO mice. Double immunofluorescence staining of pancreatic cryosections from 4 month old mice at 6 weeks after tamoxifen induction. GLP-1 receptor is expressed in both YFP^+^ and YFP^-^ insulin^+^ cells in the control islets (a-d). In beta-cell-specific *Pax6* KO islets, GLP-1 receptor expression is lost from the YFP labeled *Pax6*-deficient cells (arrowheads e-h) but maintained in the YFP^-^ insulin^+^ cells (arrows e-h).

In addition, we checked the expression of Glut2 and GLP1R, both of which play an important role in glucose stimulated insulin secretion (GSIS). Glut2 expression was lost in both the YFP labeled and unlabeled beta-cells (Fig 13), sustaining the notion that Glut2 expression is affected by hyperglycemia [30]. ChIP analysis clearly indicates that Pax6 does not appear to interact with Glut2 promoter (S4 Fig). This suggests that Pax6 may indirectly control the expression of Glut2. In contrast, GLP1R expression was lost in *Pax6*-deficient YFP-labeled cells only (Fig 14). This corroborates previous *in vitro* studies where Pax6 was shown to directly act on the expression of GLP1R [17].

**Fig 15:**
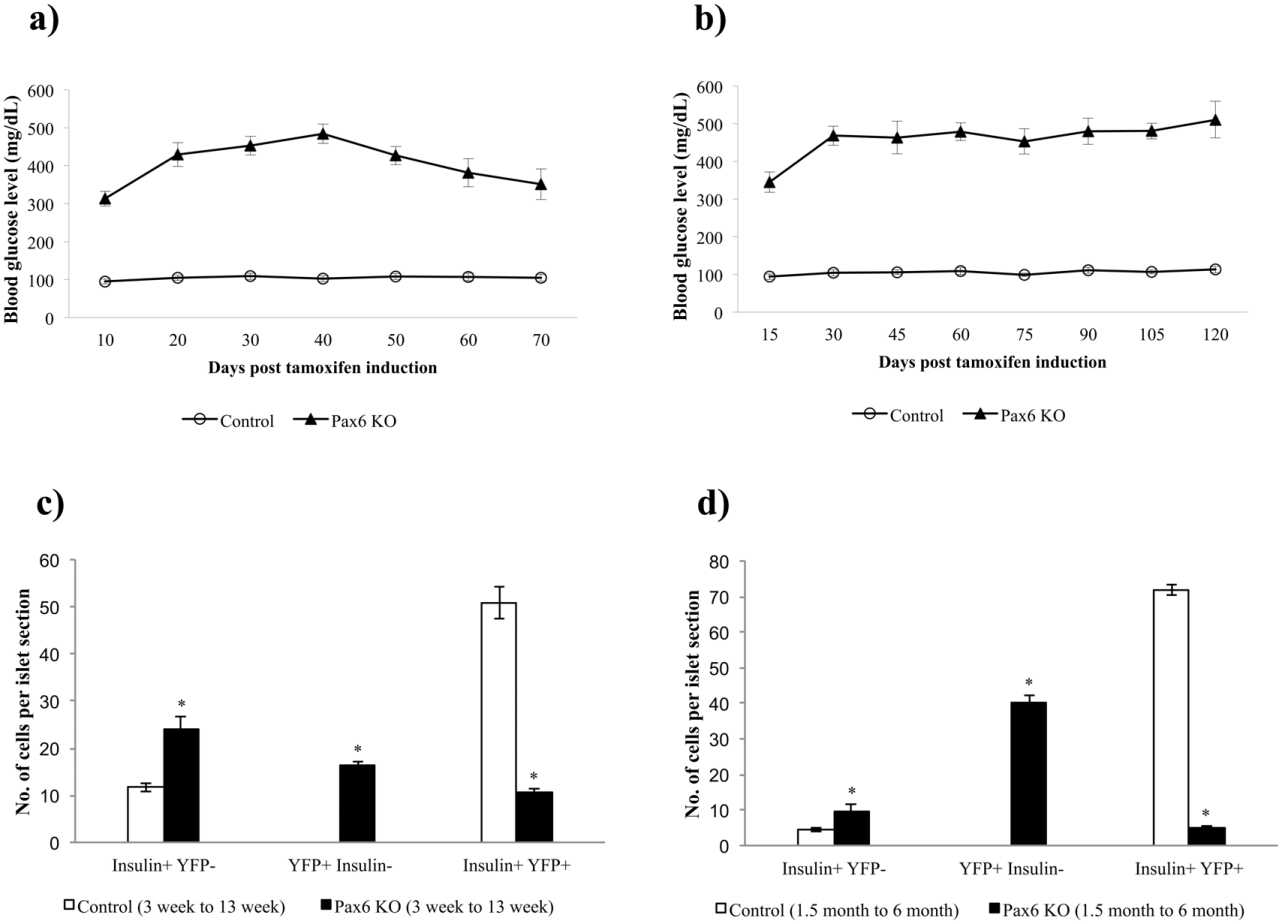
Development of hyperglycemia and resulting beta-cell regeneration in the beta-cell-specific *Pax6* KO mice. Measurement of blood glucose level in mice after tamoxifen induction at 3 weeks (a) or 1.5 month (b) (n = 5). Increase in blood glucose level is observed at either age but a slight decrease in the long term is only seen in young mice (a). Quantification of insulin^+^ YFP^-^, YFP^+^ insulin^-^, and insulin^+^ YFP^+^ cells in the islets of 13 week old mice at 10 weeks after tamoxifen induction (c) and 6 month old mice at 4.5 month after tamoxifen induction (d) (n = 3). Number of insulin^+^ YFP^-^ cells is increased at either age indicating some beta-cell regeneration. Due to the loss of insulin expression in YFP labeled *Pax6*-deficient cells, a significant increase in the number of YFP^+^ insulin^-^ and a decrease in the number of YFP^+^ insulin^+^ cells is also observed in the KO islets. (Control = *RIP-CreER;R26-YFP;Pax6* ^*+/+*^, Pax6 KO = *RIP-CreER;R26-YFP;Pax6* ^*fl/fl*^). Error bars represent SEM; *p<0.05.

Regeneration of beta-cells was shown to occur in several diabetic mouse models [21, 31, 32]. As the beta-cell-specific *Pax6* KO mice developed a sustained hyperglycemia, we were also interested whether some beta-cell regeneration may have occurred. For this purpose, we injected the mice (expressing YFP reporter) with tamoxifen at 3 weeks and 1.5 months of age and analyzed the pancreata at 10 weeks and 4.5 months post-tamoxifen induction, respectively. During this time period, the non-fasting blood glucose level was also monitored over regular intervals. In both cases, blood glucose levels started to rise until they reached a plateau. Afterwards, the blood glucose level slightly decreased in the mice that were injected at 3 weeks of age but not in those injected at 1.5 months of age (Fig 15a and 15b). Furthermore, a significant increase in the number of insulin^+^ YFP^-^ cells was observed at both ages indicating newly generated insulin^+^ cells following the KO induction. Again, this increase was more pronounced in the mice that were injected at 3 weeks of age as compared to the ones injected at 1.5 months of age (Fig 15c and 15d). This result is also in accordance with the levels of blood glucose. In conclusion, we noticed a modest and partial recovery from hyperglycemia in the mice injected at 3 weeks but not in the mice injected at 1.5 months. This is probably due to the more pronounced proliferation of beta-cells at younger age. However, in young as well as in old mice the observed increase in beta-cell number is not sufficient to allow recovery to normogylcemia.

### *Pax6*-deficient alpha-cells lose MafB and glucagon expression, while sustaining Arx expression

**Fig 16:**
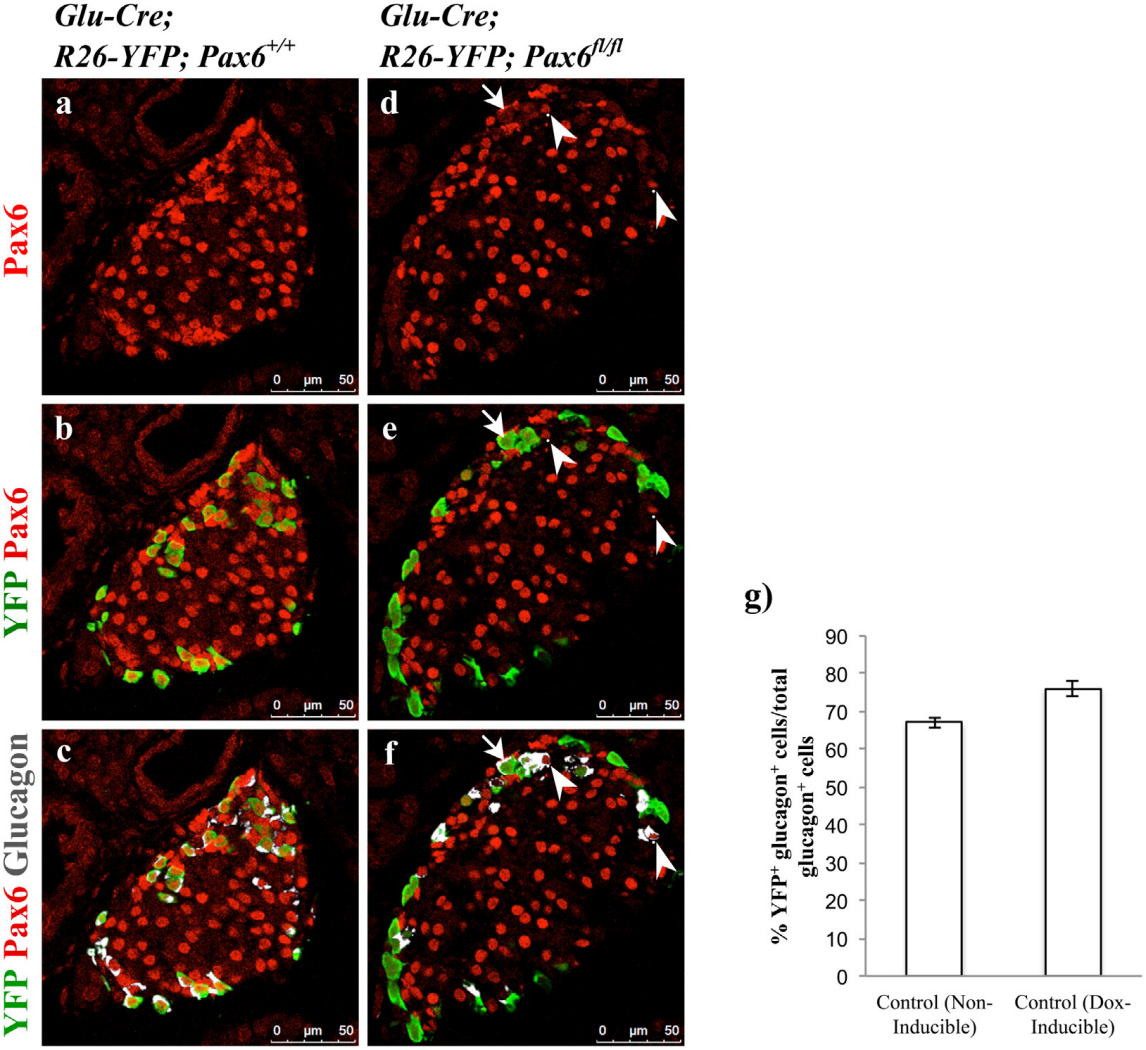
Alpha-cell-specific ablation of *Pax6*. Double immunofluorescence staining of pancreatic cryosections from 1 month old mice. In control islets, all the YFP^+^ cells express *Pax6* and glucagon (a-c). In alpha-cell-specific *Pax6* KO islets, most of the YFP^+^ cells are negative for *Pax6* and have lost the expression of glucagon as well (d-f). YFP^-^ glucagon^+^ cells in the KO islets express *Pax6* (arrowheads d-f). Rarely YFP^+^ glucagon^+^ *Pax6^-^* cells are also found in the KO islets (arrows d-f). Quantification of YFP^+^ glucagon^+^ cells in relation to total glucagon^+^ cells in 1 month old non-inducible alpha-cell-specific control (*Glu-Cre;R26-YFP;Pax6* ^*+/+*^) mice, and in Dox-inducible alpha-cell-specific control (*Glu-rtTA;TetO-Cre;R26-YFP;Pax6* ^*+/+*^) mice at 4 months of age following 6 weeks of Doxycycline treatment (g) (n = 3). Nearly 68% and 75% of the glucagon^+^ cells are labeled with YFP in non-inducible and Dox-inducible alpha-cell-specific control mice, respectively. Error bars represent SEM.

To generate alpha-cell-specific *Pax6* KO mice we used both non-inducible and tetracycline inducible Glucagon-Cre mouse lines [11, 22]. Lineage tracing was again performed in combination with *Pax6* ablation to unambiguously identify and follow the destiny of *Pax6*-deficient glucagon producing alpha-cells. Efficiency of knockout was nearly 68% in case of Glucagon-Cre and nearly 75% when using Tetracycline inducible Glucagon-Cre (Fig 16).

**Fig 17:**
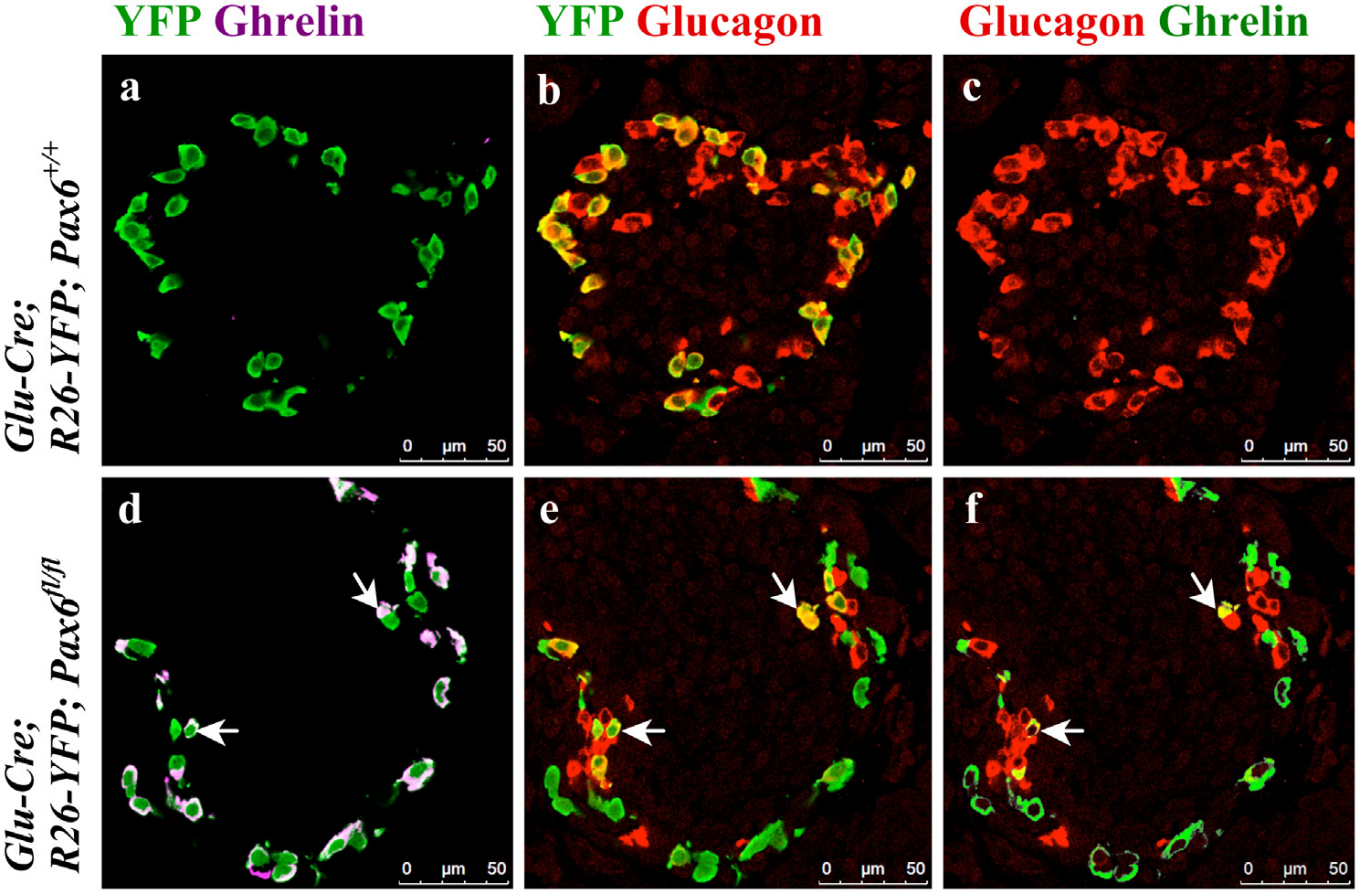
Ghrelin^+^ cells originate from alpha-cells in the alpha-cell-specific *Pax6* KO islets. Double immunofluorescence staining of pancreatic cryosections from 1 month old mice. Ghrelin expression is not detected in the control islets (a-c). In alpha-cell-specific *Pax6* KO islets ghrelin expression is up regulated in YFP labeled cells (d-f) and rarely ghrelin expression co-localizes with glucagon expression (arrows d-f).

**Fig 18:**
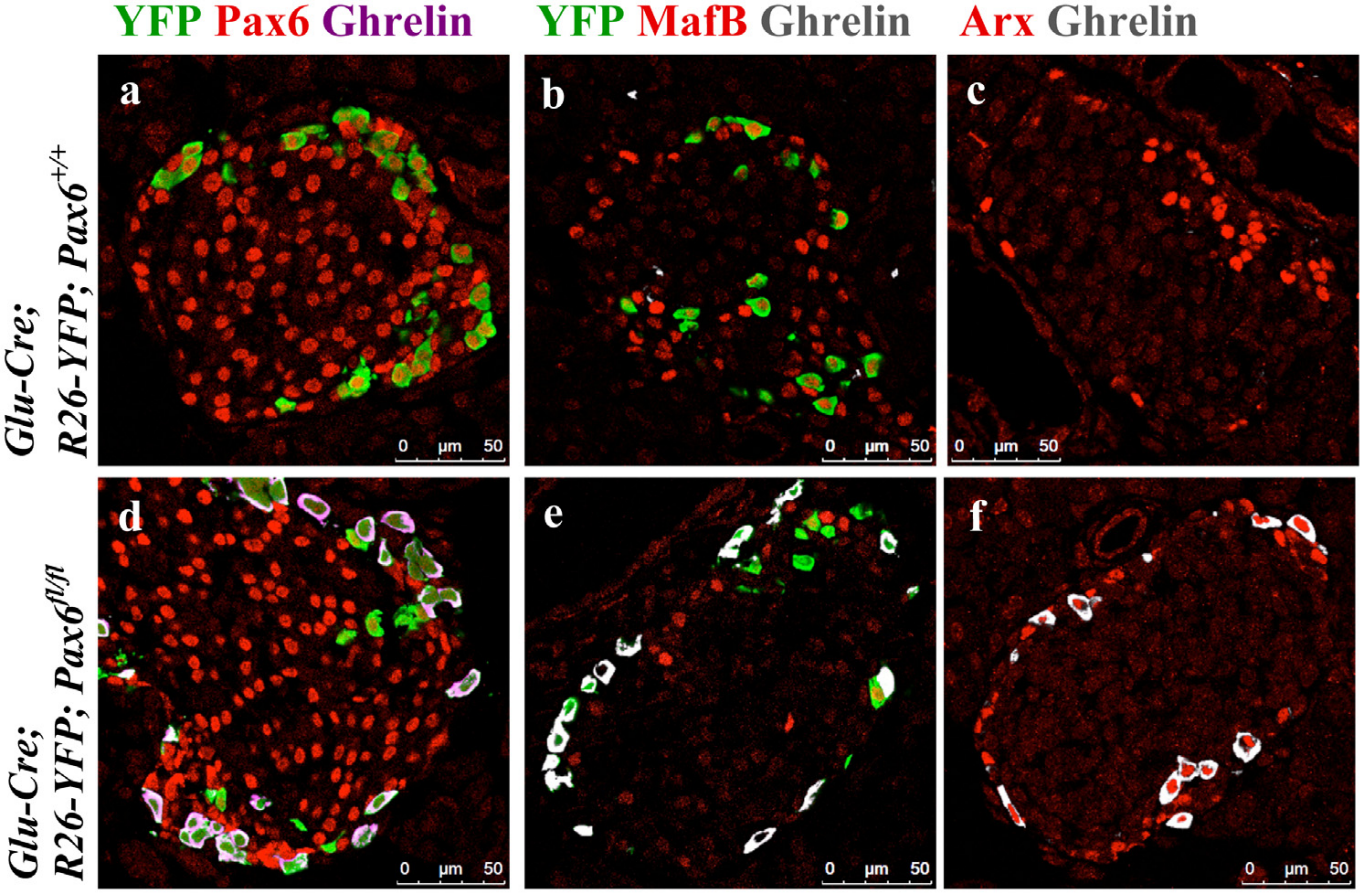
Expression of alpha-cell-related transcription factors in ghrelin^+^ cells of alpha-cell-specific *Pax6* KO islets. Double immunofluorescence staining of pancreatic cryosections from 1 month old mice. Ghrelin expression is not detected in the control islets (a-c). In alpha-cell-specific *Pax6* KO islets ghrelin expression is up regulated in YFP labeled cells and ghrelin^+^ cells are negative for Pax6 (d) and MafB (e) expression but positive for Arx (f) expression.

Our immunohistochemical analyses demonstrate that in alpha-cell-specific *Pax6* knockout pancreata, the majority of the YFP-labeled alpha-cells lost glucagon expression and become positive for ghrelin (Fig 17). Of interest was the observation that *Pax6*-deficient reporter-labeled cells were MafB^-^/Arx^+^ (Fig 18 and S5–S7 Figs). This indicates that similar to what has been found in beta-cell-specific *Pax6* knockout pancreata, the loss of *Pax6* gene activity in glucagon-producing cells provoked the erasure of alpha-cell mature differentiation characteristics, and induced the activation of increased levels of ghrelin expression. However, alpha-cells lacking Pax6 continue to express Arx and may represent alpha-cells that have lost their molecular maturation characteristics.

**Fig 19:**
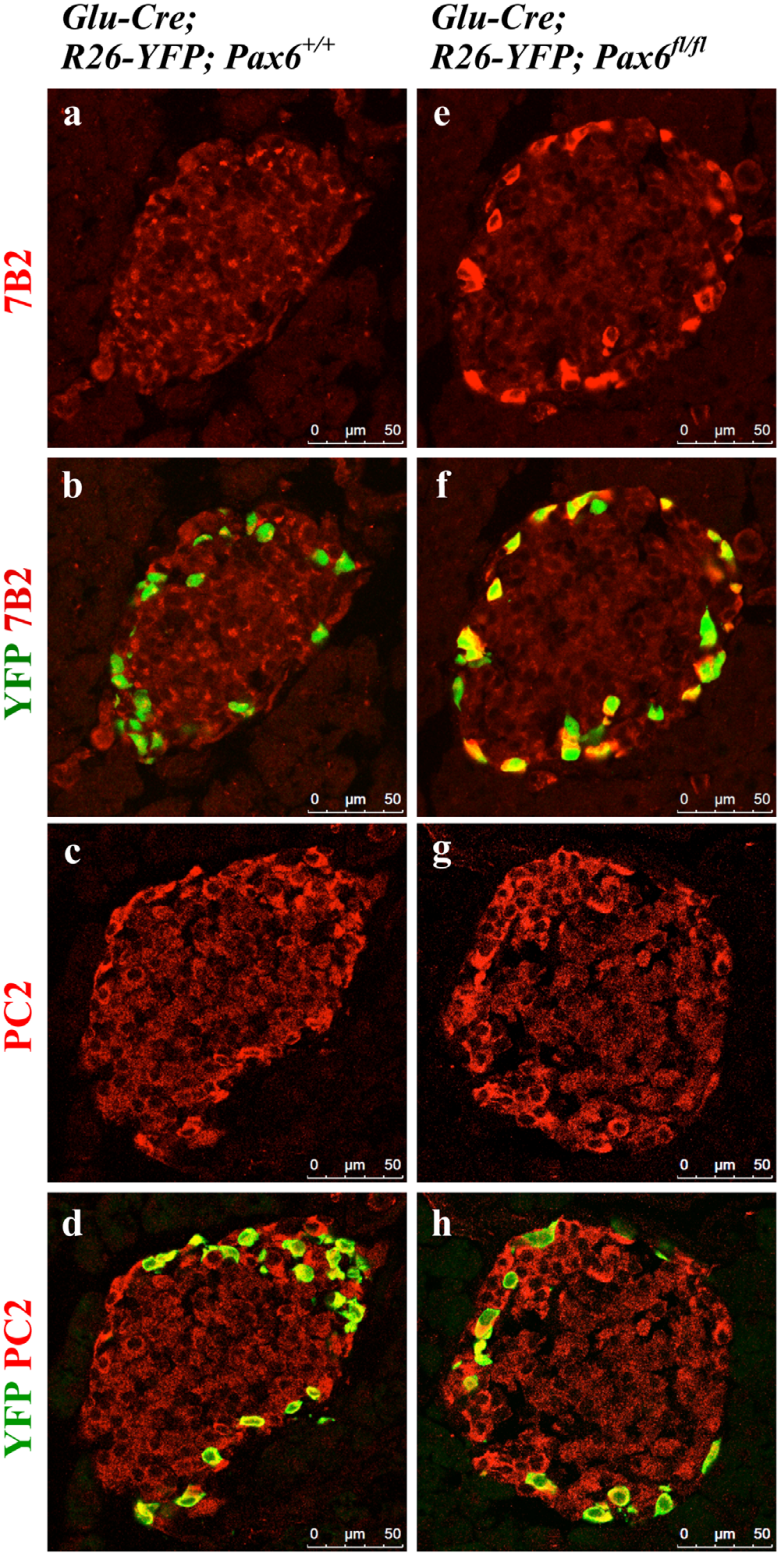
Expression of 7B2 and PC2 in alpha-cell-specific *Pax6* KO islets. Immunofluorescence staining of pancreatic cryosections from 1 month old mice. 7B2 expression is very low in the control islets (a, b). In alpha-cell-specific *Pax6* KO islets, 7B2 expression is highly up regulated in YFP labeled *Pax6*-deficient cells (e, f). Compared to control islets PC2 expression is unchanged in the alpha-cell-specific *Pax6* KO islets (c, d and g, h).

As Pax6 ablation affects insulin processing in beta-cells, we were interested to see whether this also occurs for glucagon in alpha-cells too. In YFP labeled *Pax6*-deficient alpha-cells, the expression of PC2 was preserved but that of 7B2 was highly up regulated (Fig 19).

**Fig 20:**
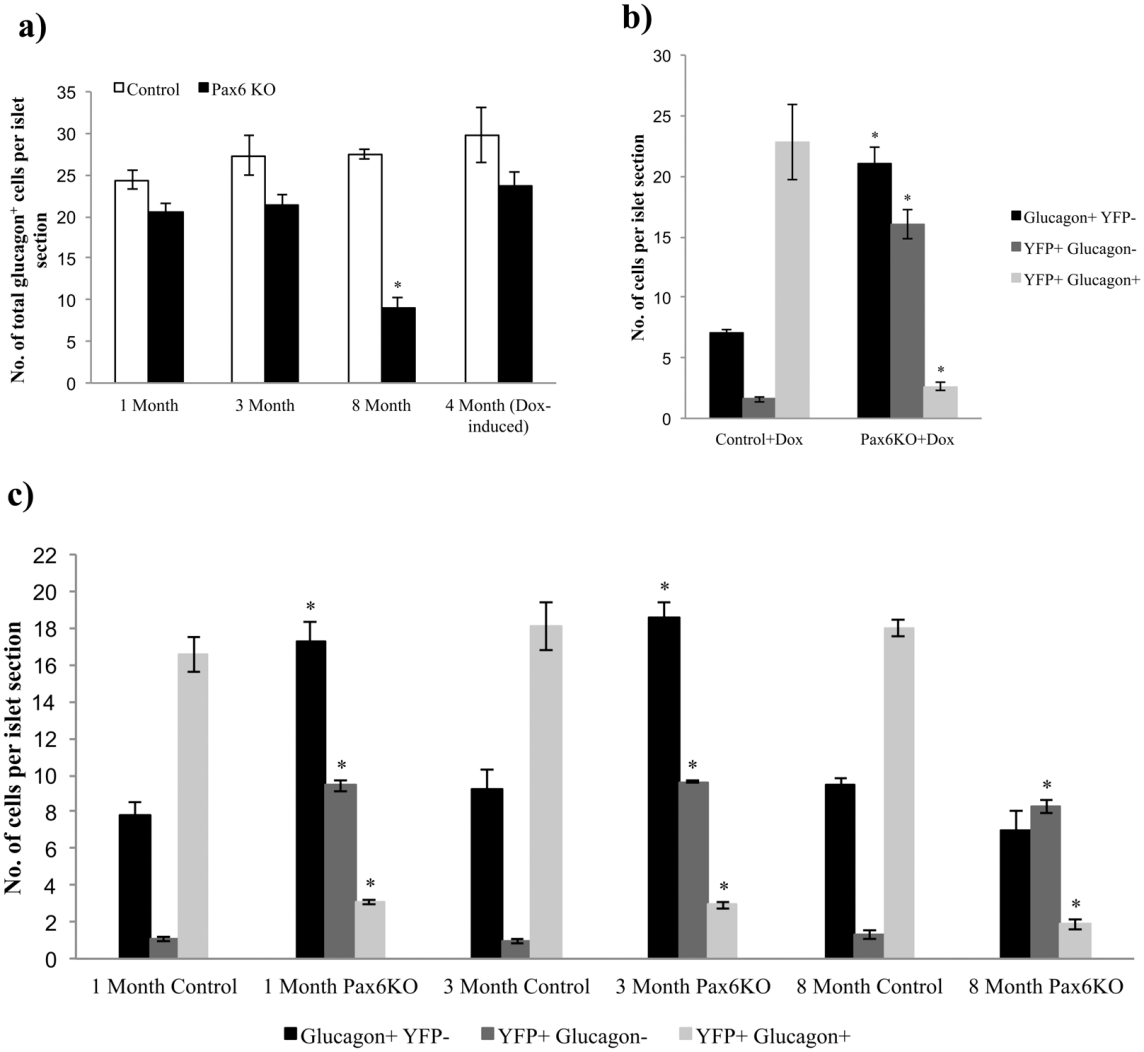
Alpha-cell neogenesis in the alpha-cell-specific *Pax6* KO islets. Quantification of total glucagon^+^ cell population in non-inducible alpha-cell-specific *Pax6* KO islets at 1 month, 3 months, and 8 months of age, and in Dox-inducible alpha-cell-specific *Pax6* KO islets at 4 months of age following 6 weeks of Doxycycline treatment (a) (n = 3). Number of total glucagon^+^ cells is unchanged at the younger age but significantly reduced at 8 months of age in the KO islets. Quantification of glucagon^+^ YFP^-^, YFP^+^ glucagon^-^, and YFP^+^ glucagon^+^ cells in Dox-inducible alpha-cell-specific *Pax6* KO islets at 4 months of age following 6 weeks of Doxycycline treatment (b) and in the non-inducible alpha-cell-specific *Pax6* KO islets at 1 month, 3 months, and 8 months of age (c) (n = 3). Number of glucagon^+^ YFP^-^ cells is significantly increased in the Dox-inducible KO islets as well as in the non-inducible KO islets at 1 month and 3 months of age but not at 8 months of age. Due to the loss of glucagon expression in YFP labeled *Pax6*-deficient cells, a significant increase in the number of YFP^+^ glucagon^-^ and a decrease in the number of YFP^+^ glucagon^+^ cells is also observed at all stages and in both types of islets. (Non-inducible control = *Glu-Cre;R26-YFP;Pax6* ^*+/+*^, Non-inducible *Pax6* KO = *Glu-Cre;R26-YFP;Pax6* ^*fl/fl*^, Dox-inducible control = *Glu-rtTA;TetO-Cre;R26-YFP; Pax6* ^*+/+*^, Dox-inducible *Pax6* KO = *Glu-rtTA;TetO-Cre;R26-YFP;Pax6* ^*fl/fl*^). Error bars represent SEM; *p<0.05.

Alpha-cell hyperplasia has been shown to occur in animals where glucagon signaling was impaired, providing strong evidence for glucagon neogenesis [9, 11, 33–35]. Given the observation that our *Pax6* glucagon-specific knockout was accompanied with the loss of glucagon expression, we were interested to examine whether the induced alpha-cell shortage may provoke alpha-cell neogenesis. Using the non-inducible Glucagon-Cre, in islets of *Glu-Cre;R26-YFP;Pax6* ^*fl/fl*^ mice at 1 or 3 month of age, the number of total glucagon^+^ cells was not significantly different from that observed in islets of control animals. However, a clear reduction of glucagon-labeled cells was detected at 8 months of age (Fig 20a). A similar phenotype was noticed when using tetracycline-inducible Glucagon-Cre, in 4 months old *Glu-rtTA;TetO-Cre;R26-YFP;Pax6* ^*fl/fl*^ mice, following 6 weeks of Doxycycline treatment (Fig 20a). This may indicate that in younger animals where glucagon cells have lost Pax6, some alpha-cell regeneration was taking place. To clarify this further, we quantified the number of YFP^-^ glucagon^+^ and YFP^+^ glucagon^+^ cells in control and KO islets. We found that the number of YFP^-^/glucagon^+^ cells was significantly increased in the KO islets at 1 month and 3 months of age but not at 8 months of age in mice from the non-inducible Glucagon-Cre line (Fig 20c). Similarly, this is also true for the *Pax6* KO islets generated using the Tetracycline-inducible Glucagon-Cre mice (Fig 20b). The significant increase in the number of such reporter negative/glucagon^+^ cells may correlate with glucagon neogenesis in the KO animals. At the same time, the number of YFP^+^ glucagon^+^ cells was significantly diminished in the KO islets, as such cells become glucagon negative upon Pax6 depletion, and further initiate the expression of ghrelin.

### *Pax6* ablation in somatostatin and PP cells of the adult pancreas

**Fig 21:**
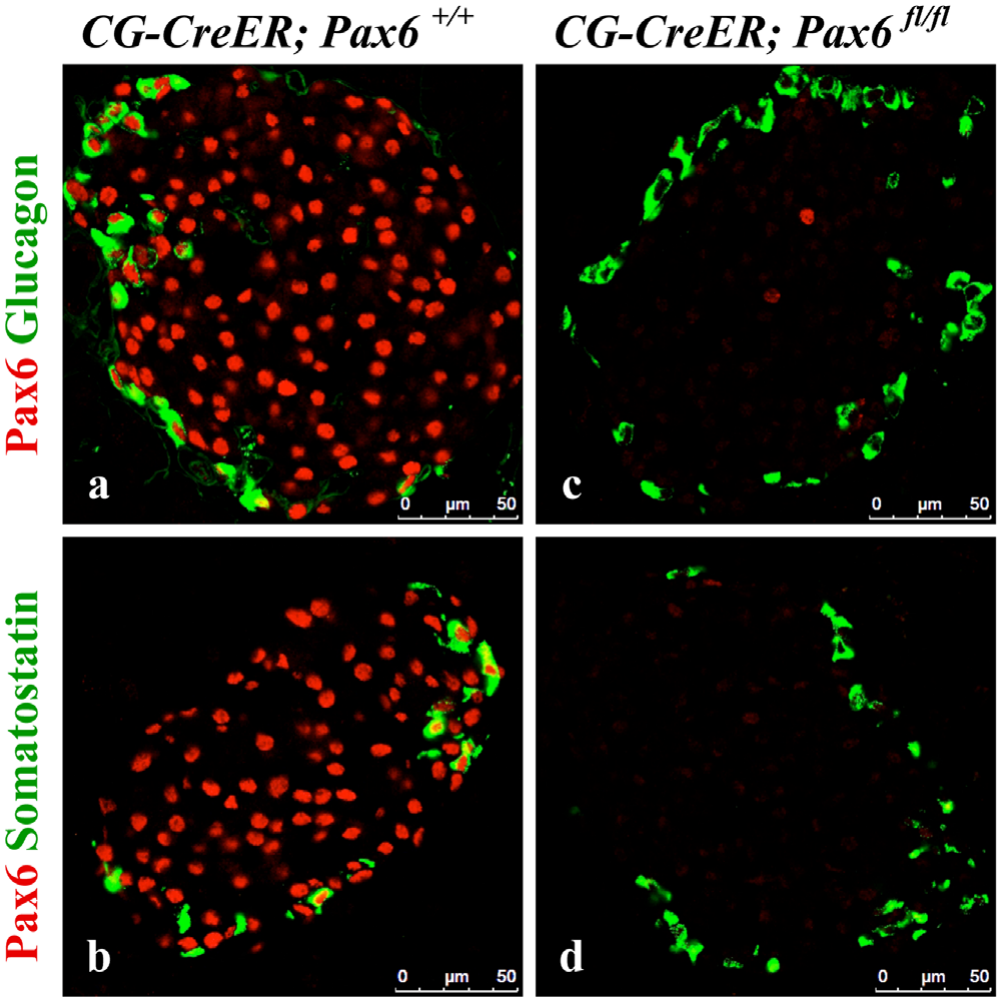
Ubiquitous ablation of *Pax6*. Double immunofluorescence staining of pancreatic cryosections from mice that were injected with tamoxifen at 2 months of age and analysed at 1 week post tamoxifen induction. A nearly 100% ablation of *Pax6* was confirmed in the islets of ubiquitous *Pax6* KO mice (c, d).

**Fig 22:**
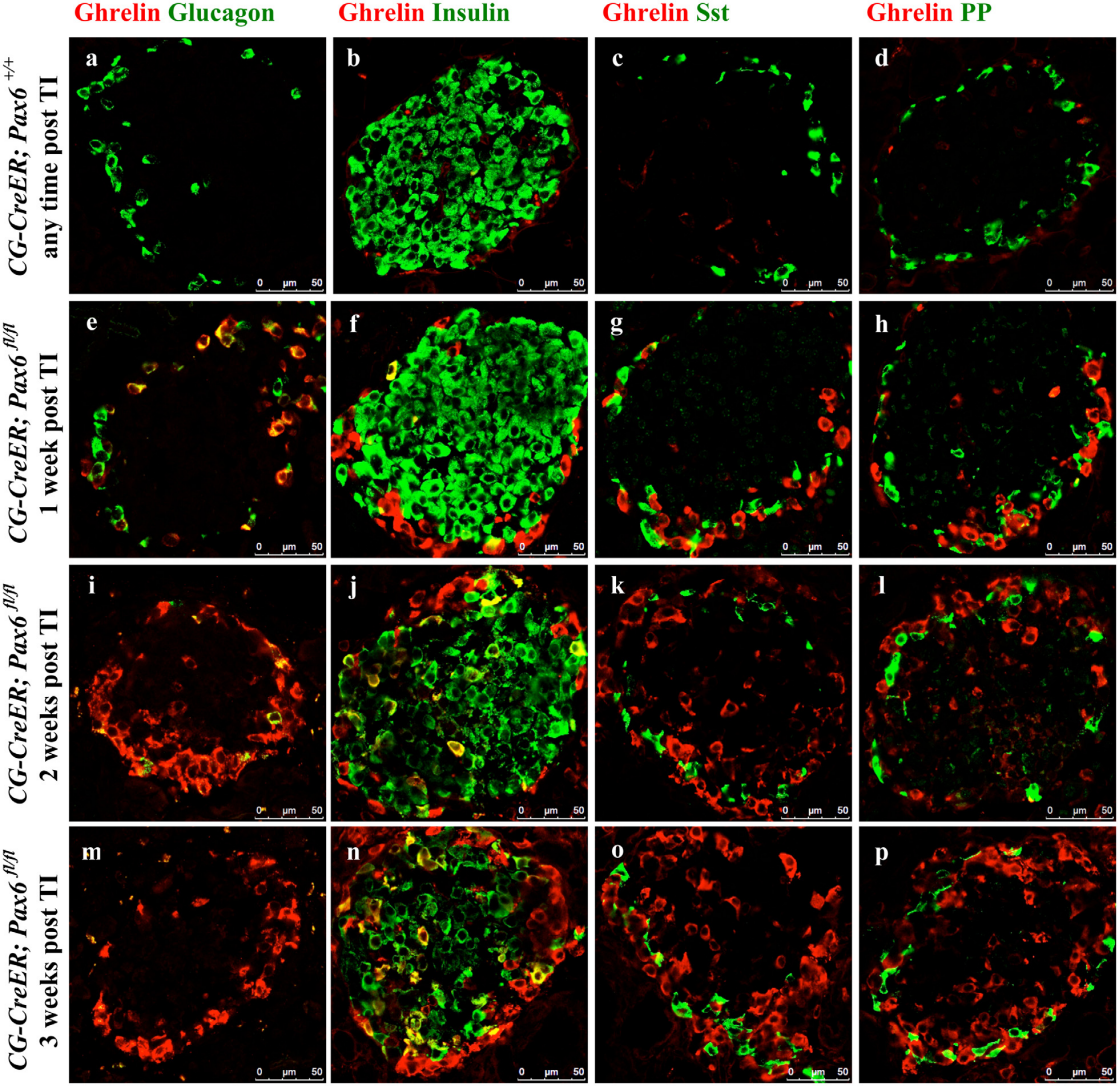
Variable effect of *Pax6* ablation in the islets of adult-ubiquitous *Pax6* KO mice. Double immunofluorescence staining of pancreatic cryosections from mice that were injected with tamoxifen at 2 months of age and analysed at 1 week, 2 weeks, and 3 weeks post tamoxifen induction. Ghrelin^+^ cells are not detected in the control islets (a-d). In the ubiquitous *Pax6* KO islets, ghrelin expression is up regulated (e-p). Ghrelin expression shows co-localization with that of glucagon (e, i, m) and insulin (f, j, n) but never with that of somatostatin (g, k, o) and PP (h, l, p). In ubiquitous *Pax6* KO islets, a loss of glucagon (e, i, m) and insulin expression (f, j, n) was observed but not that of somatostatin (g, k, o) and PP (h, l, p). Furthermore, glucagon^+^ cells were the first ones to be affected in relation to the loss of glucagon expression and upregulation of ghrelin expression (e, i, m). (TI = Tamoxifen induction).

**Fig 23:**
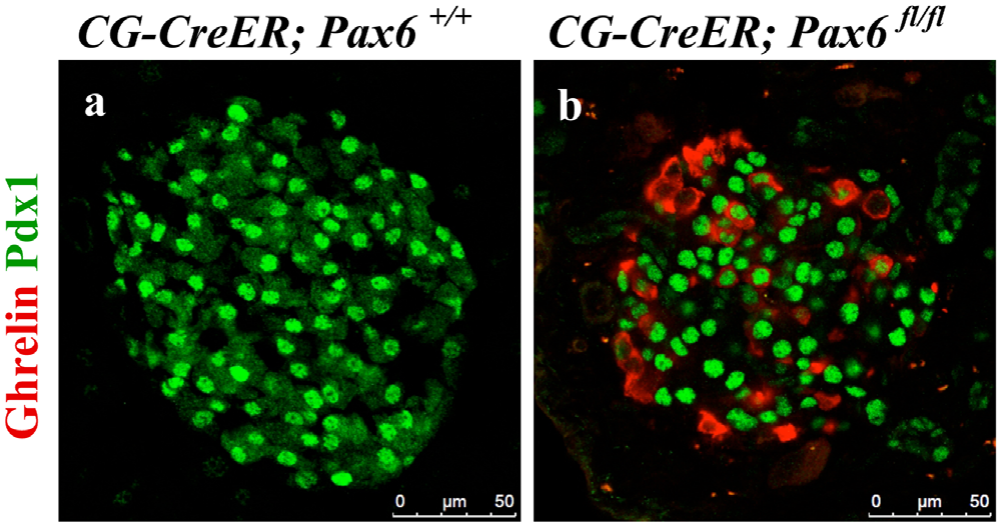
Ghrelin expressing cells, in the islets of adult-ubiquitous *Pax6* KO mice, can be differentially identified. Double immunofluorescence staining of pancreatic cryosections from mice that were injected with tamoxifen at 2 months of age and analysed at 3 weeks post tamoxifen induction. Ghrelin^+^ cells are not detected in the control islets (a). In the ubiquitous *Pax6* KO islets, ghrelin expression is up regulated and ghrelin^+^ cells may or may not express Pdx1 depending on the fact that they originally come from insulin^+^ or glucagon^+^ cells, respectively (b).

Finally, we were also interested to examine the consequences of *Pax6* ablation in somatostatin- and PP-producing cells. As no mouse line specifically expressing the Cre recombinase in delta- or PP-cells was available, we decided to use ubiquitous Cre line [25] to induce the inactivation of *Pax6* in the whole body of adult mice. Successful knockout was monitored by double immunostaining using Pax6 and glucagon/somatostatin antibodies (Fig 21). We checked the expression of ghrelin in combination with glucagon, insulin, somatostatin, and PP at 1 week, 2 week, and 3 week following tamoxifen induction. Our analyses indicate that glucagon cells appeared to be most sensitive to the loss of *Pax6* gene activity. In fact, already at 1-week post induction, most of the glucagon positive cells start to express the ghrelin hormone. Two and three week later glucagon/ghrelin co-positive cells became nearly undetectable suggesting that by that time, all of the *Pax6*-deficient alpha-cells had lost the expression of glucagon. In contrast, in beta-cells of these *Pax6* knockout mice, we noticed insulin/ghrelin co-positive cells at all the time points analysed with a slight increase in number with time. Interestingly, ghrelin positive cells expressing either somatostatin, or PP could not be detected at any stage analyzed (Fig 22). Hence, delta- and PP-cell content was not affected in these mutant islets. Furthermore, consistent with the alpha- and beta- cell specific *Pax6* ablation, the ghrelin cells appearing in these ubiquitous KO islets were also distinguishable based on the fact that they were either Pdx1^+^ (coming from beta cells) or Pdx1^-^ (coming from alpha cells) (Fig 23). It therefore appears that *Pax6* inactivation has a variable effect in different subtypes of endocrine cells of the islet. Moreover, the effect of Pax6 loss appears to be more pronounced in alpha-cells correlating with the phenotype described in global Pax6 knockout [4].

## Discussion

In the present report we studied the consequences of *Pax6* conditional inactivation in distinct endocrine cells in the adult mouse pancreas. While somatostatin- and PP-cells remained unaffected by the specific loss of *Pax6* gene activity, insulin-producing beta-cells and glucagon-labeled alpha-cells lost their maturing molecular characteristics and were shunted towards ghrelin marked cells. Based on the findings presented above it is appropriate to conclude that Pax6 is required for the maturation of alpha- and beta-cells, and Pax6 inactivation does not alter cell-subtype specification.

### *Pax6* in adult beta-cells

The use of *RIP-CreERT;Pax6* ^*fl/fl*^ mice allowed us to inactivate *Pax6* in beta-cells upon tamoxifen induction. Our findings sustain the idea that Pax6 controls the maturation of insulin producing cells. Hence, in the absence of Pax6 we could demonstrate that following tamoxifen induction two crucial beta-cell-specific markers, Glut2, and MafA were found depleted. We further noticed a gradual decrease of the expression of an additional insulin-producing cell factor, Nkx6.1. Nkx6.1 expression has been shown to be reduced in hyperglycemic *db/db* mice and T2DM human islets [36] as well as in beta-cells upon in vitro Pax6 knockdown [17]. Therefore, it seems that the Nkx6.1 expression may initially be affected by the absence of Pax6 and subsequently also by the developing hyperglycemia in these mice. In contrast, Pax6 depletion does not appear to affect *Pdx1* gene activity that remained unchanged even when followed over longer time period. Moreover, lineage-tracing experiments using *RIP-CreERT;Rosa26-YFP;Pax6* ^*fl/fl*^ mice also demonstrated that *Pax6*-deficient beta-cells undergo a progressive loss of insulin labeling which is accompanied with an activation of ghrelin expression. This is corroborated by the detection of a transient insulin^+^/ghrelin^+^ cell population. Collectively, our data provide strong evidence for the crucial role of Pax6 in beta-cell maturation. However, in the absence of Pax6 the expression of Pdx1, and Rfx6, as well as PC1/3 and IAPP persists even few months following tamoxifen induction, clearly indicating that such cells, although immature, preserve their beta-cell destiny. However, in earlier studies in cell culture (Pax6 knockdown) [17], and in mice PC1/3 and Pdx1 were found reduced [7]. On the other hand, using our Pax6 conditional knockout mice crossed with Pax6 Cre, did not reveal any change in PC1/3 expression and still displayed some Pdx1+ and Cre-labeled cells [5]. Finally, Rfx6 was recently shown to play an important role in beta-cell maturation [37, 38]. Thus, the unaltered expression of Rfx6 in *Pax6*-deficient beta-cells suggests that it acts upstream of Pax6.

In addition, it appears that Pax6 might act on cell proliferation, as documented by our lineage tracing analyses where the number of YFP-labeled beta-cells in mutant islets of older animals were significantly decreased as compared to controls, indicating that *Pax6*-deficient beta-cells may have lost the ability to proliferate over time. Pax6 binds and activates the promoter of GLP-1R [17]. Considering the role of GLP-1 in stimulating the glucose-dependent insulin secretion as well as beta-cell proliferation [39–41], a loss of GLP-1 receptor may contribute to defective insulin secretion and reduced proliferation of *Pax6*-deficient beta-cells. We therefore conclude that Pax6 may control beta-cell proliferation capacity and their maturation characteristics. Interestingly, Pax6 has been already shown to act on cell proliferation to control the pool of cell progenitors in the spinal cord [42]. On the other hand, the numbers of non-reporter labeled beta-cells (that are generated after tamoxifen induced Pax6 depletion) were increased in the mutant islets, and this augmentation was more pronounced in animals injected at younger age. In accordance with that a partial and modest recovery in hyperglycemia was also apparent in young but not in older animals. This is probably due to the increased proliferation of beta-cells at younger age.

Finally, Pax6 may act on insulin processing, as documented by the augmented expression levels of proSAAS in mutant insulin producing cells. In *Pax6*-deficient beta-cells, the expression of proSAAS (the regulatory peptide for PC1/3) and 7B2 (the regulatory peptide for PC2) were highly augmented. Increased levels of proSAAS lead to defective proinsulin processing by inhibiting the activity of PC1/3 [29]. On the other hand, 7B2 may help to rescue the remaining processing by promoting the activity of PC2 [43, 44]. The finding of increased 7B2 in our study is in contrast to a previous study where 7B2 expression was down regulated after Pax6 knockdown [14]. However, we noticed an increased 7B2 expression not only during the loss of Pax6 in beta-, but also following Pax6 depletion in alpha-cells, as well as in all endocrine cells (using ubiquitous Cre recombinase) (S8 Fig). Interestingly, both proSAAS and 7B2 may also possess prohormone convertase unrelated activities as anti-aggregation chaperones [45, 46]. Therefore, their increased expression might also be related to the deterioration of the *Pax6*-lacking cell.

### *Pax6* in alpha-cells

The ablation of P*ax6* function in glucagon-producing alpha-cells revealed a similar phenotype to what we found when inactivating *Pax6* in insulin producing beta-cells. Accordingly, the absence of Pax6 in alpha-cells provokes a gradual loss of glucagon expression that is accompanied with a depletion of MafB labeling. Similar to MafA in insulin producing beta-cells, MafB is a marker for alpha-cell maturation [47, 48]. Interestingly, the expression of the alpha-cell determination factor Arx persists in glucagon producing cells that lost *Pax6* gene activity. Thus, Pax6 does not act on Arx and therefore does not appear to promote alpha-cell specification, as suggested previously [16]. Hence, these findings suggest that, in contrast to PP- and somatostatin-expressing delta-cells, alpha-cells and beta-cells of the endocrine pancreas require *Pax6* gene activity for their maturation. This is corroborated by an earlier report using *Pax6* global knockout mice where the number of ghrelin positive cells was found increased [7]. However, in this study the fate of *Pax6*-deficient cells was not determined, and the described increase in ε-cells was not directly associated with the conversion of *Pax6*-/- cells to ghrelin labeled cells. In fact, mutant cells eventually lose insulin/glucagon expression and appear only ghrelin positive, when analysed at later time points [7]. In contrast to the previous study we have used lineage tracing to unequivocally show the conversion of *Pax6-/-* cells into ghrelin marked cells. Moreover, during the analysis of the endocrine hormones following the inactivation of *Pax6* using the ubiquitous Cre line, we could detect insulin^+^/ghrelin^+^, as well as glucagon^+^/ghrelin^+^ cells in mutant islets. Collectively, our analyses may also sustain the conclusion that ghrelin represents a marker for immature endocrine cells in the pancreas.

## Supporting Information

S1 Fig Expression of *insulin*, *ghrelin*, and beta-cell related factors in the pancreata of beta-cell-specific *Pax6* KO mice. Quantitative RT-PCR of *insulin*, *MafA*, *Nkx6*.*1*, *Glut2*, *Pdx1*, *Nkx2*.*2*, *PC1/3*, *PC2*, *and ghrelin* mRNA in the pancreata of 4.5 month old mice at 3 months after tamoxifen induction (n = 2). Error bars represent SEM; *p<0.05.
(TIF)

S2 Fig Alterations in the population of glucagon^+^, somatostatin^+^, and PP^+^ cells in the islets of beta-cell-specific *Pax6* KO mice. Quantification of glucagon^+^, somatostatin^+^, and PP^+^ cells from 2 month old mice at 4 weeks after tamoxifen induction (n = 3). Error bars represent SEM; *p<0.05.
(TIF)

S3 Fig Apoptosis is not induced in the *Pax6*-deficient cells of the beta-cell-specific *Pax6* KO islets. TUNEL staining of pancreatic cryosections from 7 week old mice at 4 weeks after tamoxifen induction. TUNEL^+^ cells are not detected in the beta-cell-specific *Pax6* KO islets (c, d).
(TIF)

S4 Fig Pax6 does not bind to *Glut2* and *ghrelin* promoters. Cross-linked chromatin from Min6 cells was precipitated by anti-Pax6 antibody and analyzed by PCR for the corresponding promoter regions. Pax6 interacts with *MafA* promoter region 3 but not with *Glut2* and *ghrelin* promoters.
(TIF)

S5 Fig Ghrelin expression is upregulated in *Pax6*-deficient cells of the alpha-cell-specific *Pax6* KO islets. Double immunofluorescence staining of pancreatic cryosections from 1 month old mice. Ghrelin expression is not detected in the YFP^+^ Pax6^+^ cells of the control islets (a-c). In alpha-cell-specific *Pax6* KO islets ghrelin expression is upregulated in YFP labeled *Pax6*-deficient cells (d-f).
(TIF)

S6 Fig Expression of alpha-cell related transcription factors in ghrelin^+^ cells of alpha-cell-specific *Pax6* KO islets. Double immunofluorescence staining of pancreatic cryosections from 1 month old mice. Ghrelin expression is not detected in the control islets (a-c). In alpha-cell-specific *Pax6* KO islets ghrelin expression is upregulated in YFP labeled cells and ghrelin^+^ cells are negative for MafB expression (d-f). Rarely some YFP^-^ ghrelin^+^ cells are also detected in the KO islets and they are also negative for MafB (d-f).
(TIF)

S7 Fig Expression of alpha-cell related transcription factors in ghrelin^+^ cells of alpha-cell-specific *Pax6* KO islets. Double immunofluorescence staining of pancreatic cryosections from 1 month old mice. Ghrelin expression is not detected in the control islets (a-c). In alpha-cell-specific *Pax6* KO islets ghrelin expression is upregulated in YFP labeled cells and ghrelin^+^ cells are positive for Arx expression (d-f). Rarely some YFP^-^ ghrelin^+^ cells are also detected in the KO islets and they are also positive for Arx (d-f).
(TIF)

S8 Fig Gradual increase in the population of ghrelin and 7B2 expressing cells in the islets of adult-ubiquitous *Pax6* KO mice. Double immunofluorescence staining of pancreatic cryosections from mice that were injected with tamoxifen at 2 months of age and analysed at 1 week and 3 weeks post tamoxifen induction. Ghrelin^+^ cells are not detected and the expression of 7B2 is very low in the control islets (a-c). At 1 week after tamoxifen induction few cells start to express 7B2 and ghrelin at a higher level in the KO islets (d-f). At 3 weeks after tamoxifen induction a large number of cells express high levels of 7B2 and ghrelin in the KO islets (g-i). 7B2 expression in the KO islets may or may not co-localize with ghrelin expression (d-i). (TI = tamoxifen induction).
(TIF)

S1 Text Supplementary materials and methods. (DOC)
