# Supplementary figures and images for: Pax6 Inactivation in the Adult Pancreas Reveals Ghrelin as Endocrine Cell Maturation Marker
Source: PLoS One. 2015 Dec 11;10(12):e0144597. doi: 10.1371/journal.pone.0144597 (PMC4676685; doi:10.1371/journal.pone.0144597)

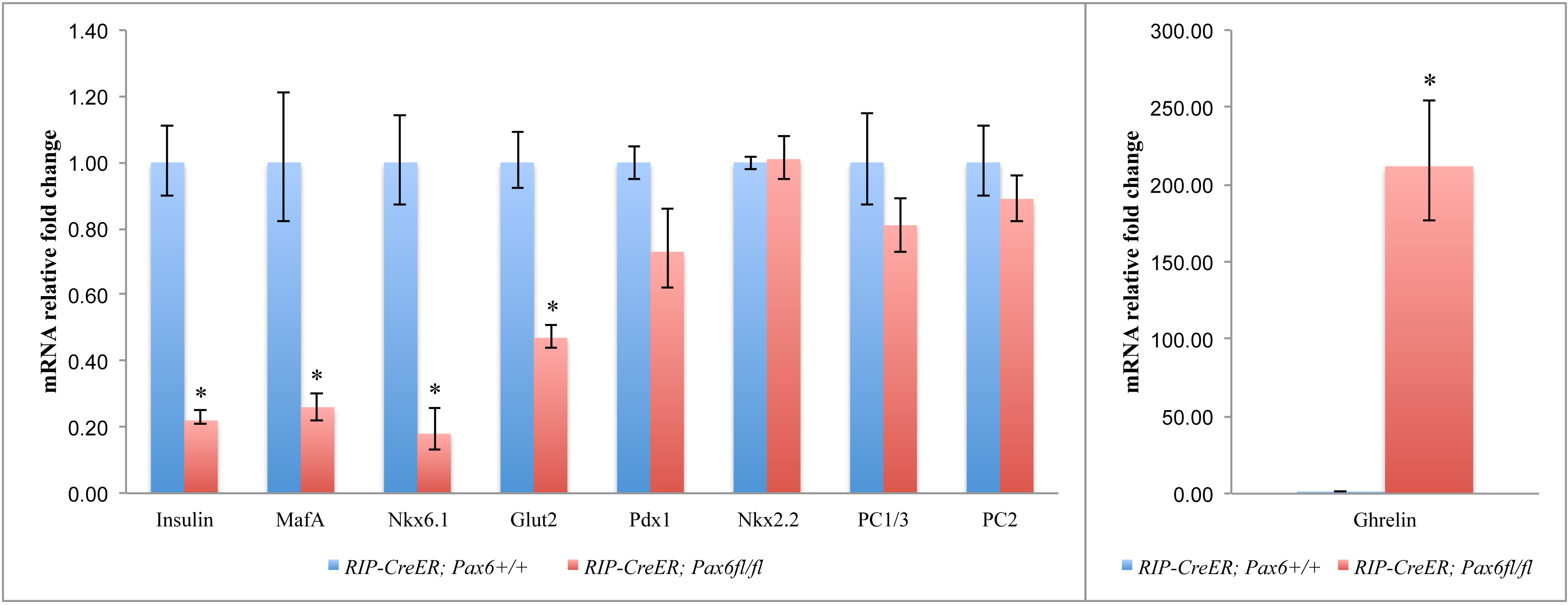

Supplement: S1 Fig — Quantitative RT-PCR of insulin, MafA, Nkx6.1, Glut2, Pdx1, Nkx2.2, PC1/3, PC2, and ghrelin mRNA in the pancreata of 4.5 month old mice at 3 months after tamoxifen induction (n = 2). Error bars represent SEM; *p<0.05. (TIF) [file pone.0144597.s001.tif]

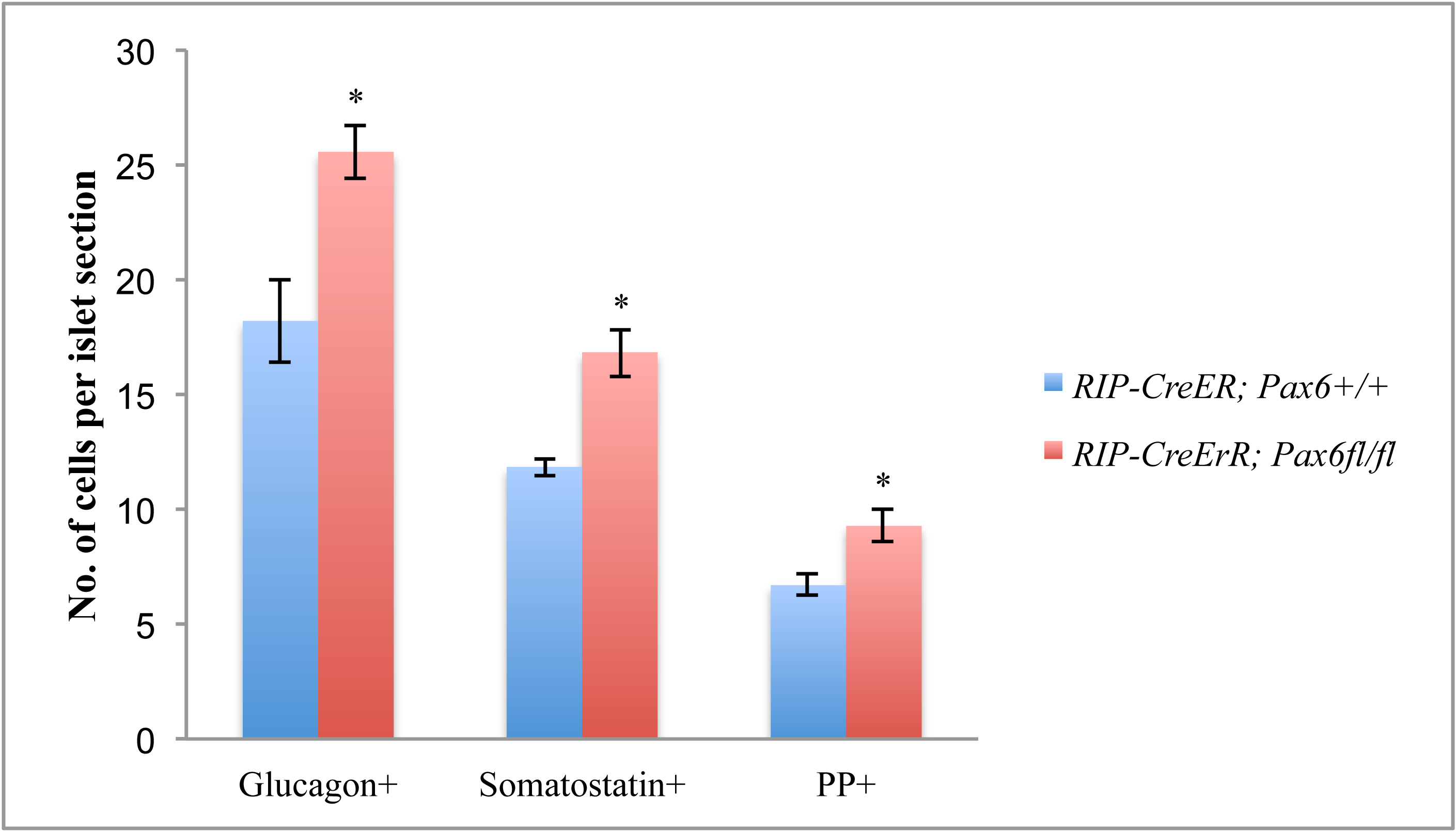

Supplement: S2 Fig — Quantification of glucagon+, somatostatin+, and PP+ cells from 2 month old mice at 4 weeks after tamoxifen induction (n = 3). Error bars represent SEM; *p<0.05. (TIF) [file pone.0144597.s002.tif]

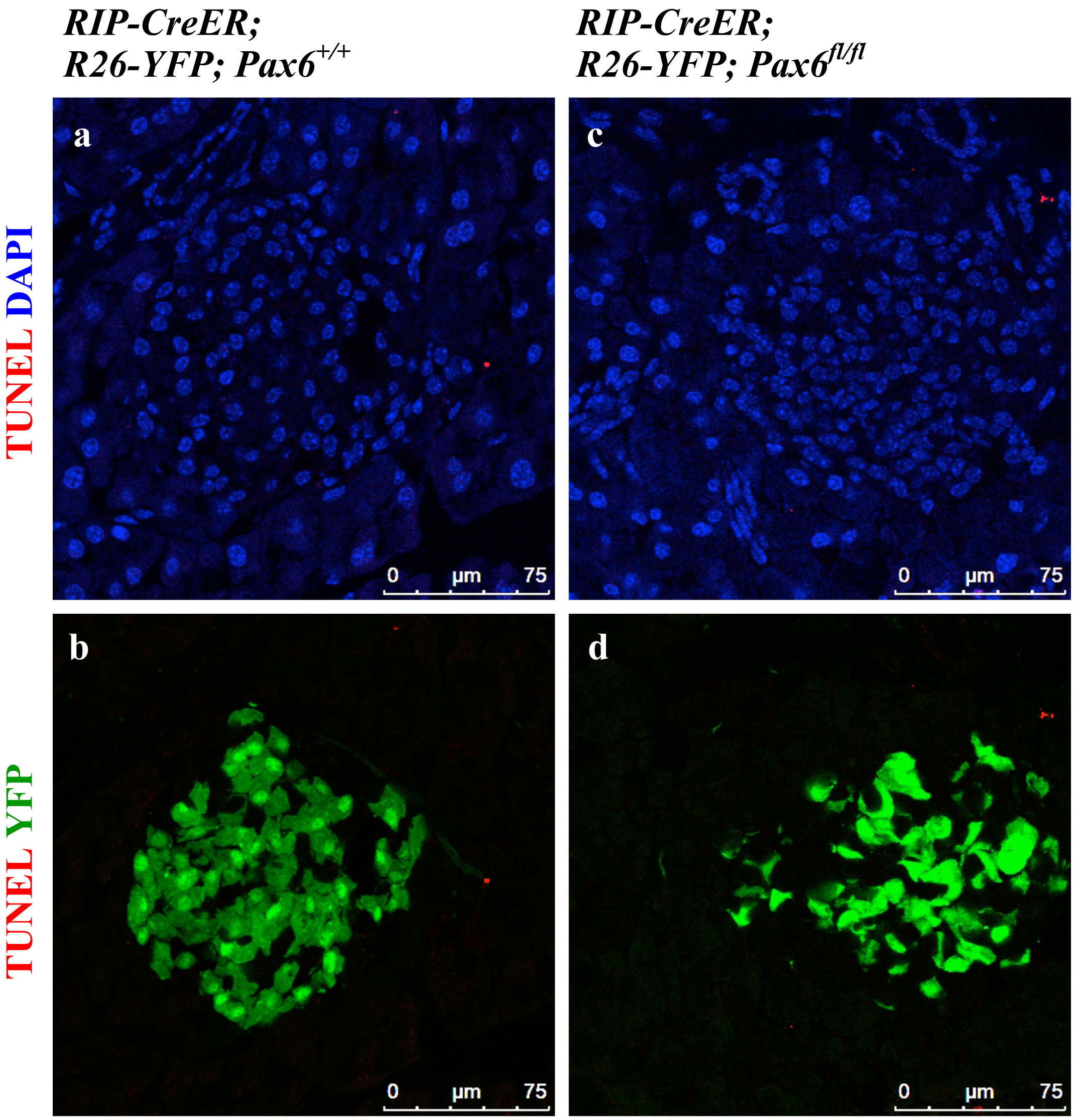

Supplement: S3 Fig — TUNEL staining of pancreatic cryosections from 7 week old mice at 4 weeks after tamoxifen induction. TUNEL+ cells are not detected in the beta-cell-specific Pax6 KO islets (c, d). (TIF) [file pone.0144597.s003.tif]

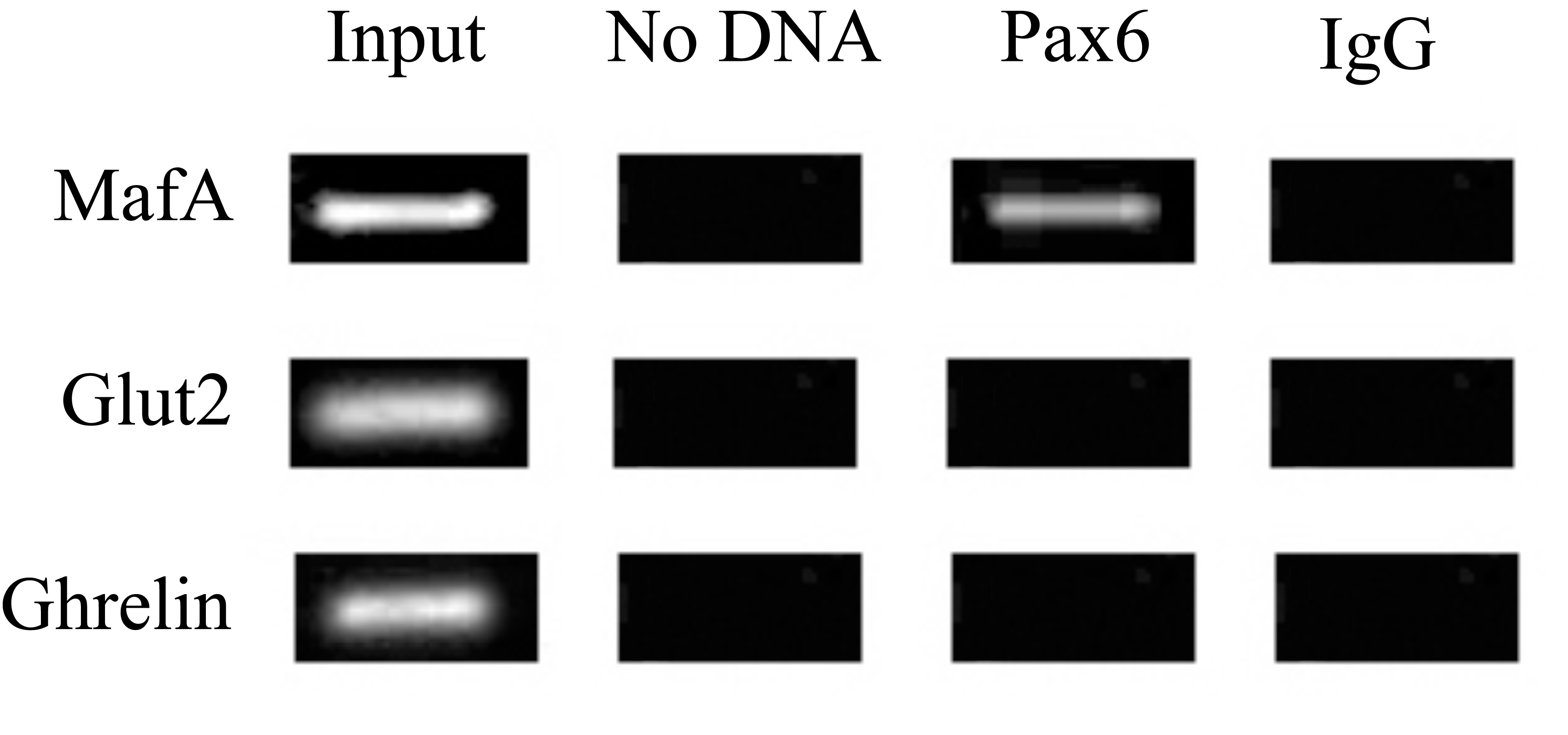

Supplement: S4 Fig — Cross-linked chromatin from Min6 cells was precipitated by anti-Pax6 antibody and analyzed by PCR for the corresponding promoter regions. Pax6 interacts with MafA promoter region 3 but not with Glut2 and ghrelin promoters. (TIF) [file pone.0144597.s004.tif]

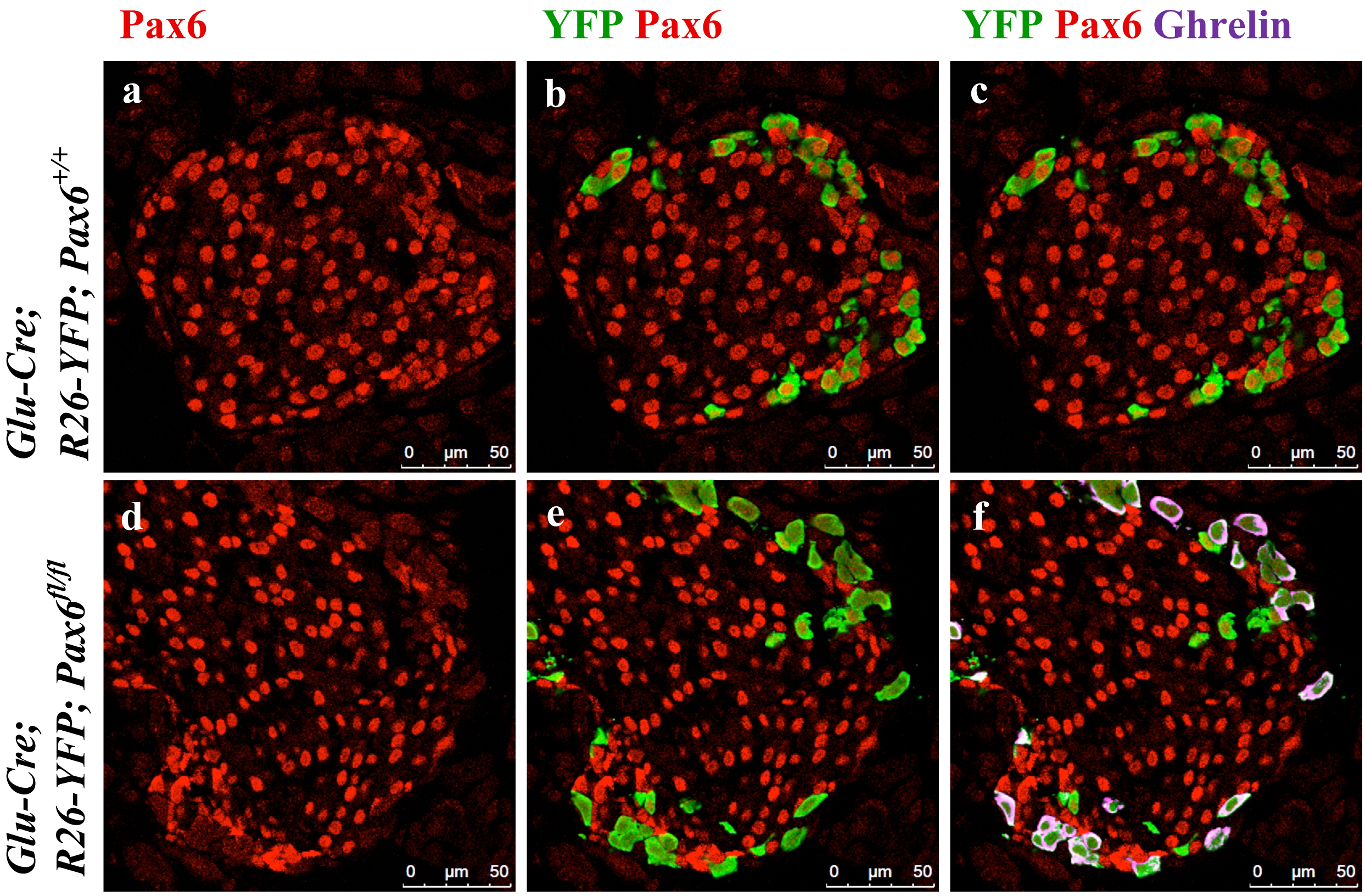

Supplement: S5 Fig — Double immunofluorescence staining of pancreatic cryosections from 1 month old mice. Ghrelin expression is not detected in the YFP+ Pax6+ cells of the control islets (a-c). In alpha-cell-specific Pax6 KO islets ghrelin expression is upregulated in YFP labeled Pax6-deficient cells (d-f). (TIF) [file pone.0144597.s005.tif]

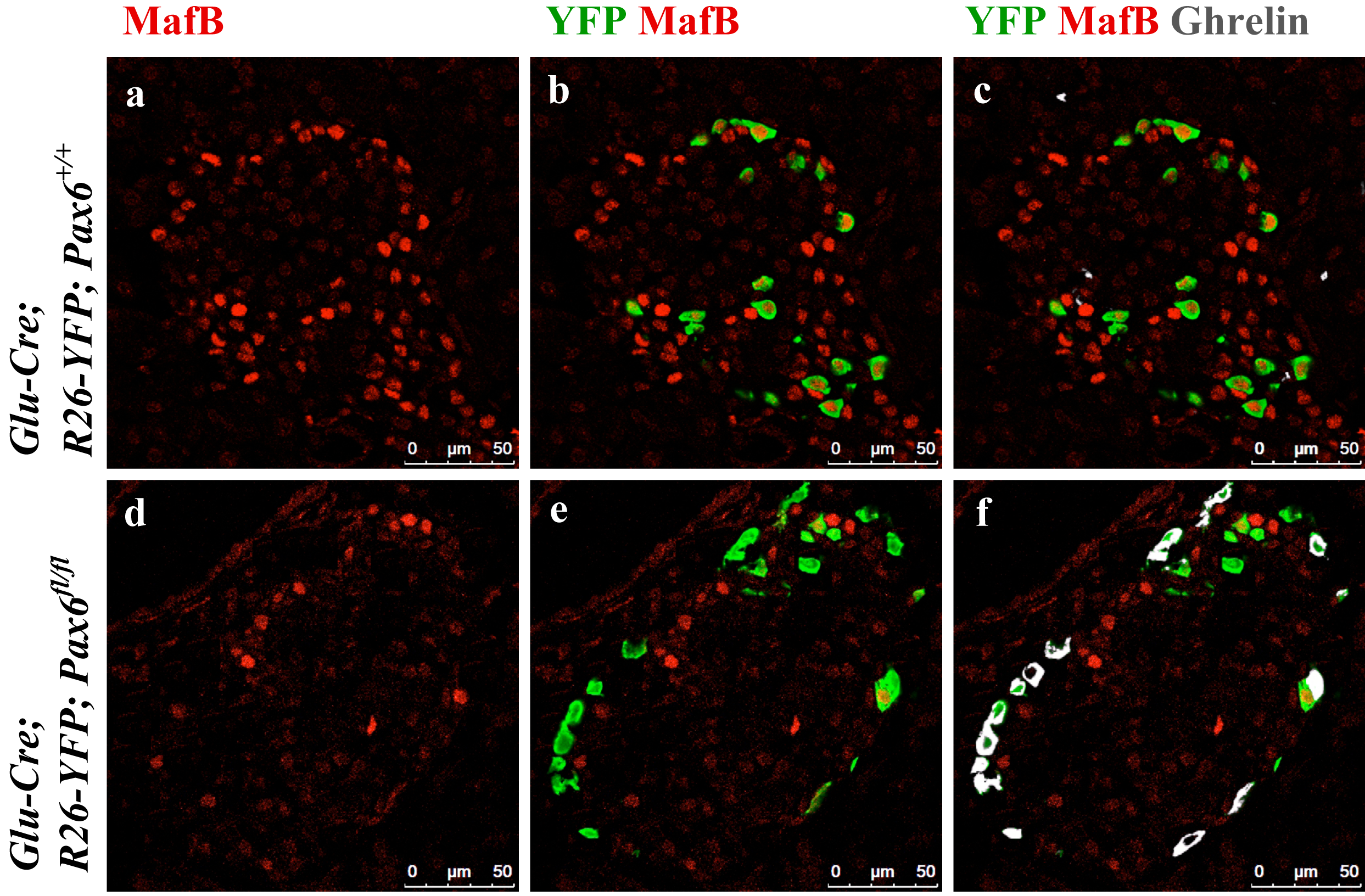

Supplement: S6 Fig — Double immunofluorescence staining of pancreatic cryosections from 1 month old mice. Ghrelin expression is not detected in the control islets (a-c). In alpha-cell-specific Pax6 KO islets ghrelin expression is upregulated in YFP labeled cells and ghrelin+ cells are negative for MafB expression (d-f). Rarely some YFP- ghrelin+ cells are also detected in the KO islets and they are also negative for MafB (d-f). (TIF) [file pone.0144597.s006.tif]

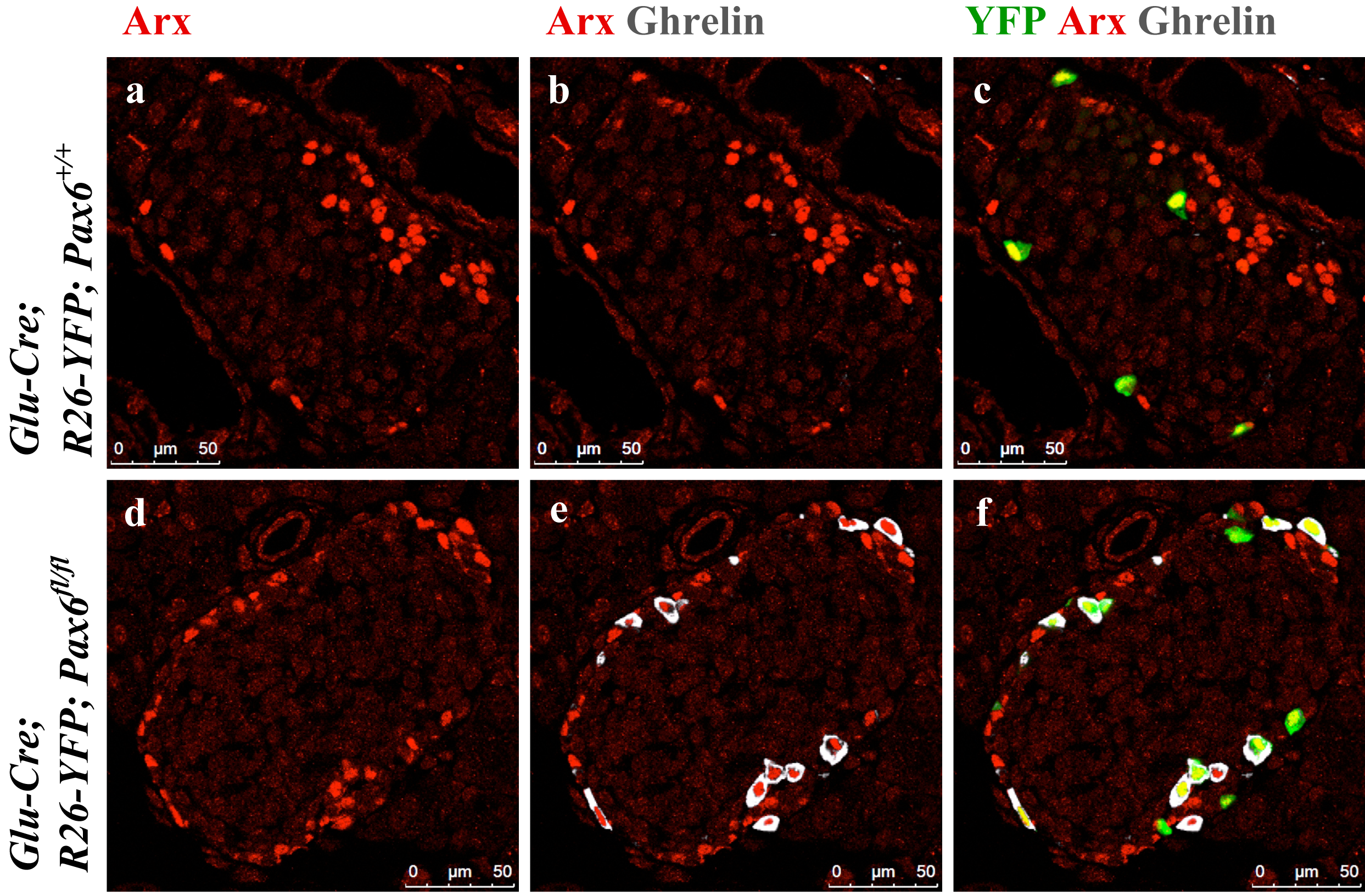

Supplement: S7 Fig — Double immunofluorescence staining of pancreatic cryosections from 1 month old mice. Ghrelin expression is not detected in the control islets (a-c). In alpha-cell-specific Pax6 KO islets ghrelin expression is upregulated in YFP labeled cells and ghrelin+ cells are positive for Arx expression (d-f). Rarely some YFP- ghrelin+ cells are also detected in the KO islets and they are also positive for Arx (d-f). (TIF) [file pone.0144597.s007.tif]

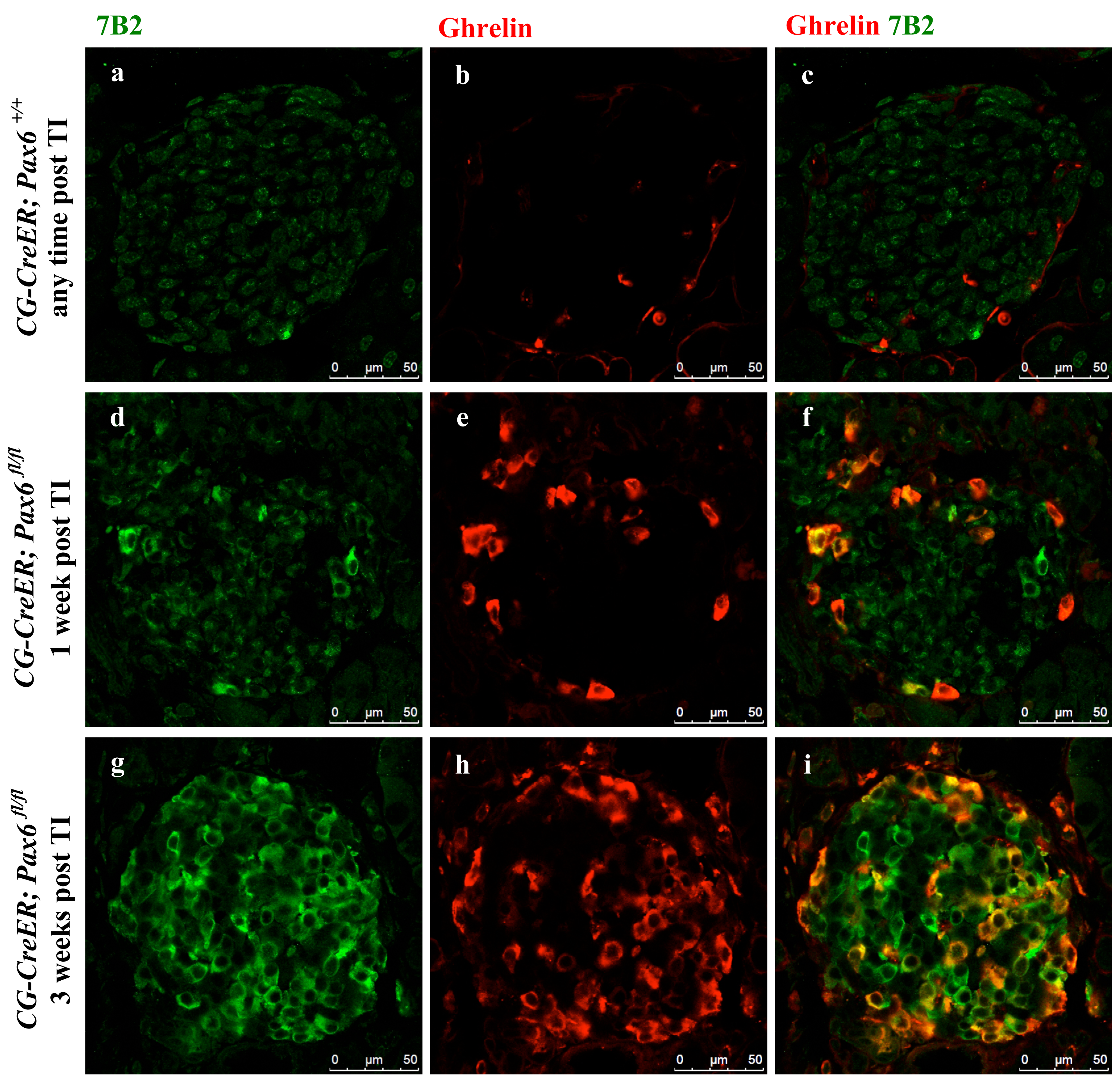

Supplement: S8 Fig — Double immunofluorescence staining of pancreatic cryosections from mice that were injected with tamoxifen at 2 months of age and analysed at 1 week and 3 weeks post tamoxifen induction. Ghrelin+ cells are not detected and the expression of 7B2 is very low in the control islets (a-c). At 1 week after tamoxifen induction few cells start to express 7B2 and ghrelin at a higher level in the KO islets (d-f). At 3 weeks after tamoxifen induction a large number of cells express high levels of 7B2 and ghrelin in the KO islets (g-i). 7B2 expression in the KO islets may or may not co-localize with ghrelin expression (d-i). (TI = tamoxifen induction). (TIF) [file pone.0144597.s008.tif]
