# Supplementary material for: Pax6 Inactivation in the Adult Pancreas Reveals Ghrelin as Endocrine Cell Maturation Marker
Source: PLoS One. 2015 Dec 11;10(12):e0144597. doi: 10.1371/journal.pone.0144597 (PMC4676685; doi:10.1371/journal.pone.0144597)
Supplement: S1 Text — (DOC) [file pone.0144597.s009.doc]

**Supplementary materials and methods**

*Quantitative real-time PCR (qRT-PCR)*

Pancreata were collected in RNA*later* (Qiagen) and stored at 4°C for overnight. Next day, the pancreatic tissue was disrupted by means of Tissuelyser (Qiagen). Total RNA was then extracted from the lysate using RNeasy Plus Mini Kit (Qiagen) and converted to cDNA with the help of QuantiTect Reverse Transcription Kit (Qiagen) according to the manufacturer’s instructions. Finally, quantitative real-time PCR was performed, using the QuantiTect SYBR Green PCR Kit (Qiagen), in Mastercycler® realplex2 (Eppendorf). The primers used are enlisted in Table S1. *Beta-glucuronidase* (Gusb) was used as an internal control. Relative expression was quantified using the 2^-ΔΔCT^ method (1). Standard error of the mean was calculated for the ΔCT values and converted into the fold change to show in the final graph. Students’s t-test was applied and P<0.05 was considered significant.

Table S1: List of primers used in qRT-PCR

| Gene | Primer |
| --- | --- |
| *Gusb* | QuantiTect Primer Assay QT00176715 |
| *Insulin* | QuantiTect Primer Assay QT00258083 |
| *Ghrelin* | QuantiTect Primer Assay QT00137536 |
| *MafA* | QuantiTect Primer Assay QT01037638 |
| *Glut2* | QuantiTect Primer Assay QT00103537 |
| *Nkx6.1* | QuantiTect Primer Assay QT00143318 |
| *Nkx2.2* | QuantiTect Primer Assay QT00495502 |
| *Pdx1* | QuantiTect Primer Assay QT00102235 |
| *PC1/3* | Minami et al., 2005 (2) |
| *PC2* | Minami et al., 2005 (2) |

*TUNEL staining*

Apoptosis was checked by performing TUNEL (terminal deoxynucleotidyl transferase dUTP nick end labeling) staining on pancreatic cryosections. For this purpose, Apop Tag® Red In Situ Apoptosis Detection Kit (Millipore) was used according to the manufacturer’s instructions.

*Chromatin immunoprecipitation (ChIP) assay*

ChIP assay was performed on Min6 cells using EZ-ChIP^TM^ kit (Millipore) according to the manufacturer’s instructions. Briefly, formaldehyde was added directly to the culture medium (final concentration- 1%) to cross-link chromatin in 90% confluent Min6 cells. Cross-linking was carried out at room temperature for 10 minutes and the reaction was stopped by adding glycine to the medium (final concentration-125 mM) and incubating for additional 5 minutes at room temperature. Subsequent steps were performed on ice. Cells were washed in PBS and resuspended in 1mL lysis buffer containing protease inhibitor cocktail. Cross-linked DNA was then sheared by sonication to produce a fragment length of ≈200-1000 base pairs. Following sonication the samples were centrifuged and supernatant was diluted 10 times in dilution buffer containing protease inhibitor cocktail. Protein G Agarose beads were used to pre-clear the diluted chromatin for 1 hour at 4°C on a rotating platform. From the pre-cleared chromatin, 1% was saved as input and the rest was incubated with 10 μg of rabbit anti-Pax6 antibody (Covance) or 10 μg of normal rabbit IgG (Millipore). After overnight incubation at 4°C on a rotating platform, the antibody-protein-DNA complexes were collected by adding Protein G Agarose beads and incubating for 1 hour at 4°C with rotation. The beads were then washed through a series of wash buffers and complexes were eluted with elution buffer. Lastly, the protein-DNA crosslinks were reversed and the DNA was purified.

MatInspector (<http://www.genomatix.de/>) was used to find in silico Pax6 binding sites in *Glut2* and *ghrelin* promoters. Based on this result primers were designed (Table S2). PCR was performed to check if Pax6 does really bind to *ghrelin* and *Glut2* promoters. PCR for Pax6 binding site in *MafA* promoter region 3 was included as a positive control (3).

**Table S2: List of primers used to amplify ChIP eluted DNA**

| Primer | Sequence | Promoter-region amplified |
| --- | --- | --- |
| Glut2-ChIP-F | CCTAAGACACAGAAAAGTCACAGGG | -415 to -547 (contains proposed Pax6 binding site) |
| Glut2-ChIP-R | GTGGCCACAGAGTGTGGCAGCATCG |
| Ghrelin-ChIP-F | GGAGAAGCCGGTGAGCAGGCACCAC | -335 to -476 (contains proposed Pax6 binding site) |
| Ghrelin-ChIP-R | CTGAATAATTTAGACCCCGGTGAGC |
| MafA-ChIP-F | Raum et al., 2010 (3) | -7750 to -8120 |
| MafA-ChIP-R |

**Supplementary references**

1. [Livak KJ](http://www.ncbi.nlm.nih.gov/pubmed/?term=Livak KJ%5BAuthor%5D&cauthor=true&cauthor_uid=11846609), [Schmittgen TD](http://www.ncbi.nlm.nih.gov/pubmed/?term=Schmittgen TD%5BAuthor%5D&cauthor=true&cauthor_uid=11846609).

Analysis of relative gene expression data using real-time quantitative PCR and the 2(-Delta Delta C(T)) Method. Methods. 2001 Dec;25(4):402-8.

2. [Minami K](http://www.ncbi.nlm.nih.gov/pubmed/?term=Minami K%5BAuthor%5D&cauthor=true&cauthor_uid=16210247), [Okuno M](http://www.ncbi.nlm.nih.gov/pubmed/?term=Okuno M%5BAuthor%5D&cauthor=true&cauthor_uid=16210247), [Miyawaki K](http://www.ncbi.nlm.nih.gov/pubmed/?term=Miyawaki K%5BAuthor%5D&cauthor=true&cauthor_uid=16210247), [Okumachi A](http://www.ncbi.nlm.nih.gov/pubmed/?term=Okumachi A%5BAuthor%5D&cauthor=true&cauthor_uid=16210247), [Ishizaki K](http://www.ncbi.nlm.nih.gov/pubmed/?term=Ishizaki K%5BAuthor%5D&cauthor=true&cauthor_uid=16210247), [Oyama K](http://www.ncbi.nlm.nih.gov/pubmed/?term=Oyama K%5BAuthor%5D&cauthor=true&cauthor_uid=16210247), [Kawaguchi M](http://www.ncbi.nlm.nih.gov/pubmed/?term=Kawaguchi M%5BAuthor%5D&cauthor=true&cauthor_uid=16210247), [Ishizuka N](http://www.ncbi.nlm.nih.gov/pubmed/?term=Ishizuka N%5BAuthor%5D&cauthor=true&cauthor_uid=16210247), [Iwanaga T](http://www.ncbi.nlm.nih.gov/pubmed/?term=Iwanaga T%5BAuthor%5D&cauthor=true&cauthor_uid=16210247), [Seino S](http://www.ncbi.nlm.nih.gov/pubmed/?term=Seino S%5BAuthor%5D&cauthor=true&cauthor_uid=16210247). Lineage tracing and characterization of insulin-secreting cells generated from adult pancreatic acinar cells. Proc Natl Acad Sci U S A. 2005;102(42):15116-21. Epub 2005 Oct 6.

3. [Raum JC](http://www.ncbi.nlm.nih.gov/pubmed/?term=Raum JC%5BAuthor%5D&cauthor=true&cauthor_uid=20584984), [Hunter CS](http://www.ncbi.nlm.nih.gov/pubmed/?term=Hunter CS%5BAuthor%5D&cauthor=true&cauthor_uid=20584984), [Artner I](http://www.ncbi.nlm.nih.gov/pubmed/?term=Artner I%5BAuthor%5D&cauthor=true&cauthor_uid=20584984), [Henderson E](http://www.ncbi.nlm.nih.gov/pubmed/?term=Henderson E%5BAuthor%5D&cauthor=true&cauthor_uid=20584984), [Guo M](http://www.ncbi.nlm.nih.gov/pubmed/?term=Guo M%5BAuthor%5D&cauthor=true&cauthor_uid=20584984), [Elghazi L](http://www.ncbi.nlm.nih.gov/pubmed/?term=Elghazi L%5BAuthor%5D&cauthor=true&cauthor_uid=20584984), [Sosa-Pineda B](http://www.ncbi.nlm.nih.gov/pubmed/?term=Sosa-Pineda B%5BAuthor%5D&cauthor=true&cauthor_uid=20584984), [Ogihara T](http://www.ncbi.nlm.nih.gov/pubmed/?term=Ogihara T%5BAuthor%5D&cauthor=true&cauthor_uid=20584984), [Mirmira RG](http://www.ncbi.nlm.nih.gov/pubmed/?term=Mirmira RG%5BAuthor%5D&cauthor=true&cauthor_uid=20584984), [Sussel L](http://www.ncbi.nlm.nih.gov/pubmed/?term=Sussel L%5BAuthor%5D&cauthor=true&cauthor_uid=20584984), [Stein R](http://www.ncbi.nlm.nih.gov/pubmed/?term=Stein R%5BAuthor%5D&cauthor=true&cauthor_uid=20584984). Islet beta-cell-specific MafA transcription requires the 5'-flanking conserved region 3 control domain. Mol Cell Biol. 2010 (17):4234-44. doi: 10.1128/MCB.01396-09. Epub 2010 Jun 28.
